# Two-way Automated Text Messaging Support From Community Pharmacies for Medication Taking in Multiple Long-term Conditions: Human-Centered Design With Nominal Group Technique Development Study

**DOI:** 10.2196/41735

**Published:** 2022-12-21

**Authors:** Gemma Donovan, Nicola Hall, Felicity Smith, Jonathan Ling, Scott Wilkes

**Affiliations:** 1 Generated Health London United Kingdom; 2 University of Sunderland Sunderland United Kingdom; 3 School of Pharmacy University College London London United Kingdom; 4 Faculty of Health Sciences and Wellbeing University of Sunderland Sunderland United Kingdom; 5 School of Medicine University of Sunderland Sunderland United Kingdom

**Keywords:** medication adherence, text messaging, human-centered design, complex interventions, community pharmacy

## Abstract

**Background:**

Reviews of digital communication technologies suggest that they can be effective in supporting medication use; however, their use alongside nondigital components is unclear. We also explored the delivery of a digital communication intervention in a relatively novel setting of community pharmacies and how such an intervention might be delivered to patients with multiple long-term conditions. This meant that despite the large number of intervention examples available in the literature, design questions remained, which we wanted to explore with key stakeholders. Examples of how to involve stakeholders in the design of complex health care interventions are lacking; however, human-centered design (HCD) has been suggested as a potential approach.

**Objective:**

This study aimed to design a new community pharmacy text messaging intervention to support medication use for multiple long-term conditions, with patient and health care professional stakeholders in primary care.

**Methods:**

HCD was used to map the intervention “journey” and identify design questions to explore with patients and health care professionals. Six prototypes were developed to communicate the intervention concept, and a modified version of the Nominal Group Technique was used to gather feedback. Nominal group meetings generated qualitative data using questions about the aspects that participants liked for each prototype and any suggested changes. The discussion was analyzed using a framework approach to transform feedback into statements. These statements were then ranked using a web-based questionnaire to establish a consensus about what elements of the design were valued by stakeholders and what changes to the design were most important.

**Results:**

A total of 30 participants provided feedback on the intervention design concept over 5 nominal group meetings (21 health care professionals and 9 patients) with a 57% (17/30) response rate to the ranking questionnaire. Furthermore, 51 proposed changes in the intervention were generated from the framework analysis. Of these 51 changes, 27 (53%) were incorporated into the next design stage, focusing on changes that were ranked highest. These included suggestions for how text message content might be tailored, patient information materials, and the structure for pharmacist consultation. All aspects that the participants liked were retained in the future design and provided evidence that the proposed intervention concept had good acceptability.

**Conclusions:**

HCD incorporating the Nominal Group Technique is an appropriate and successful approach for obtaining feedback from key stakeholders as part of an iterative design process. This was particularly helpful for our intervention, which combined digital and nondigital components for delivery in the novel setting of a community pharmacy. This approach enabled the collection and prioritization of useful multiperspective feedback to inform further development and testing of our intervention. This model has the potential to minimize research waste by gathering feedback early in the complex intervention design process.

## Introduction

### Background

Medication nonadherence is a known problem internationally, with 30%-50% of patients not taking medicines as prescribed, particularly in the case of long-term conditions [1]. This means that patients do not attain the health gains expected from medication and represent an avoidable cost to health care systems [2]. Patients are also increasingly managing multiple long-term conditions (MLTCs) [3], resulting in the requirement to manage multiple medicines. This poses additional challenges for patients [4].

Evidence suggests that digital communication technologies can improve medication adherence [5]; however, most interventions are not designed for patients with MLTCs [6]. Methods to develop such interventions are lacking, but updated guidance on complex intervention development from the Medical Research Council (MRC) suggests that approaches such as human-centered design (HCD) could be helpful [7]. Some reviews of digital communication to improve medication adherence have also suggested that their use may be optimized when delivered alongside other components such as face-to-face consultations or telephone appointments [8-10]. However, the contribution of these additional components to overall effectiveness is unclear [6].

### Intervention Description

This study describes our first phase of development of a community pharmacy–delivered text messaging intervention to support medication use for patients with MLTCs. Our setting is in the United Kingdom National Health Service (NHS), where approximately 99% of community pharmacies have an NHS contract to deliver health care services [11,12]. This includes the New Medicines Service [13,14] to support medication taking and, at the time of this project, Medicines Use Reviews [15,16], although these have since been decommissioned.

Our proposed intervention concept aimed to combine an automated two-way text messaging program with community pharmacy support. Our program theory used concepts from the Behavior Change Wheel [17] and was developed using intervention mapping [18]. The Behavior Change Wheel includes the Capability, Opportunity, and Motivation model (COM-B) for behavior performance, with each component able to affect behavior. Our program theory centered on how COM-B is applied to the behavior of “taking medication” to influence the outcome of medication adherence. Similar to others [19], we also considered medication adherence to be an outcome that exists in the spectrum between “suboptimal” and “increased” adherence levels.

**Figure 1:**
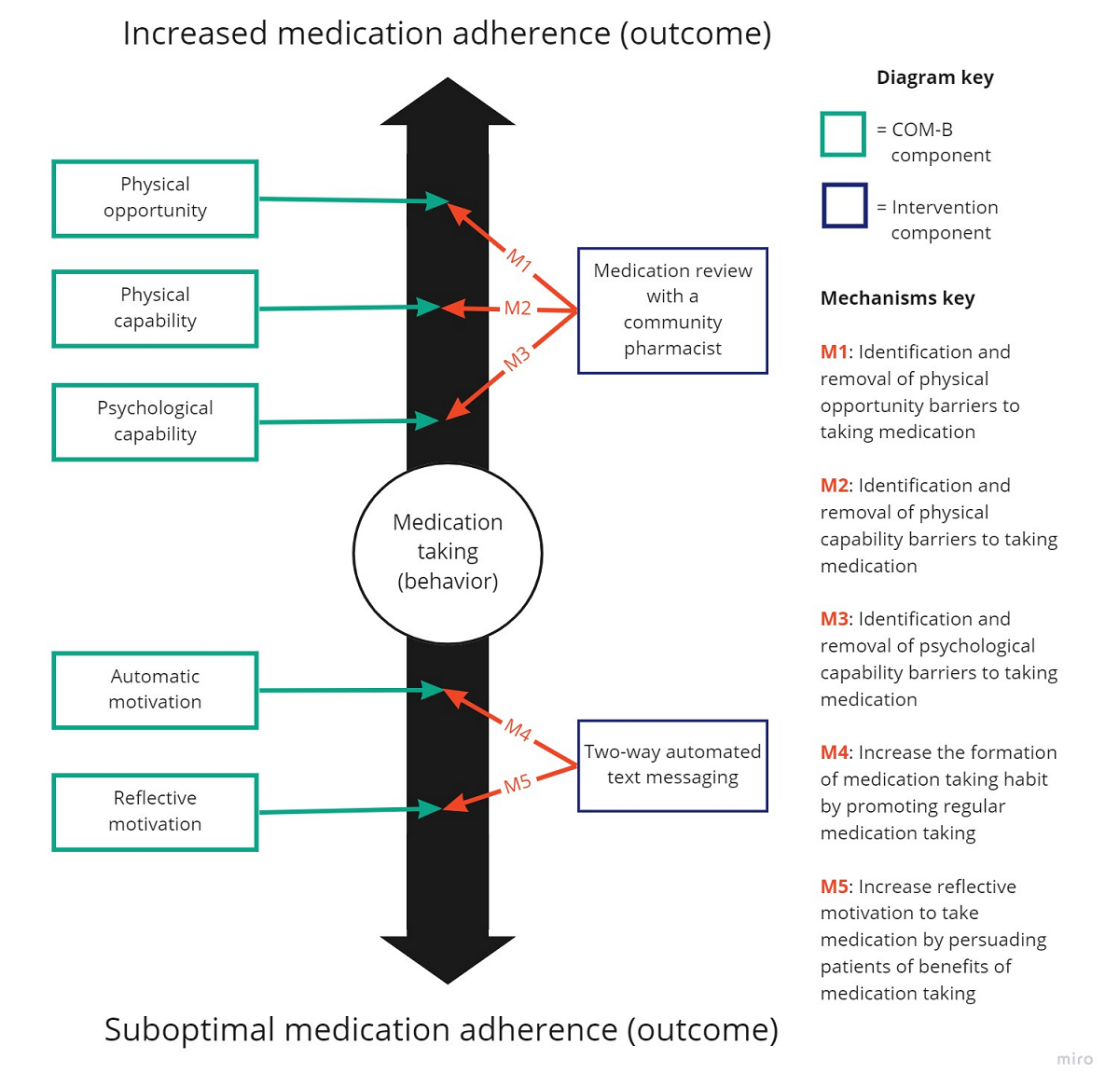
Program theory for how the new intervention combining 2-way automated text messaging delivered from a community pharmacy would work to support medication taking.

Physical opportunity, physical capability, and psychological capability for taking medication by patients were intended to be assessed during a face-to-face pharmacist consultation, with barriers resolved by either the patient or pharmacist, depending on the barriers identified. Two-way automated text messaging aims to support habit formation via the Automatic Motivation component of the COM-B and influence Reflective Motivation for taking medication. An overview of the program theory can be found in Figure 1 and was developed based on our systematic review [6] and experience as health care professionals (HCPs), with input from a project steering group that included patients. However, as examples of similar interventions were lacking and the intervention included both digital and nondigital components, we felt that an HCD design process would be helpful in further developing the intervention concept.

### Intervention Design Approach

Evidence from a systematic review [20] that informed MRC recommendations on the development of complex interventions suggests that the strength of HCD is its focus on patient experience. However, examples of how prototypes, a key feature of HCD, can support complex health care intervention designs are lacking. Our approach used the HCD toolkit developed by IDEO.org [21], where appropriate tools are recommended for selection depending on the individual intervention. In this study, we describe how we used prototyping to involve patients and HCPs in the intervention design.

We used the guidance for reporting intervention development studies in health research checklist [22] to construct this study, and a copy of the completed checklist is available in Multimedia Appendix 1. This paper aimed to both provide readers with an understanding of our proposed intervention and an example approach for using HCD and prototypes to develop complex health care interventions.

## Methods

### Overview

We used the HCD toolkit from IDEO.org [21] to guide the iterative design process and incorporated a modified version of Nominal Group Technique (NGT) [23] to gather and analyze feedback from patient and HCP participants. NGT was chosen to explore consensus on the most important aspects that participants liked and what changes were most important. This consensus was anticipated to be important for informing design changes in a scenario where it would be unfeasible to make all changes or where there may be incompatible changes suggested between participants, which would require resolution.

### HCD and Prototype Development

**Figure 2:**
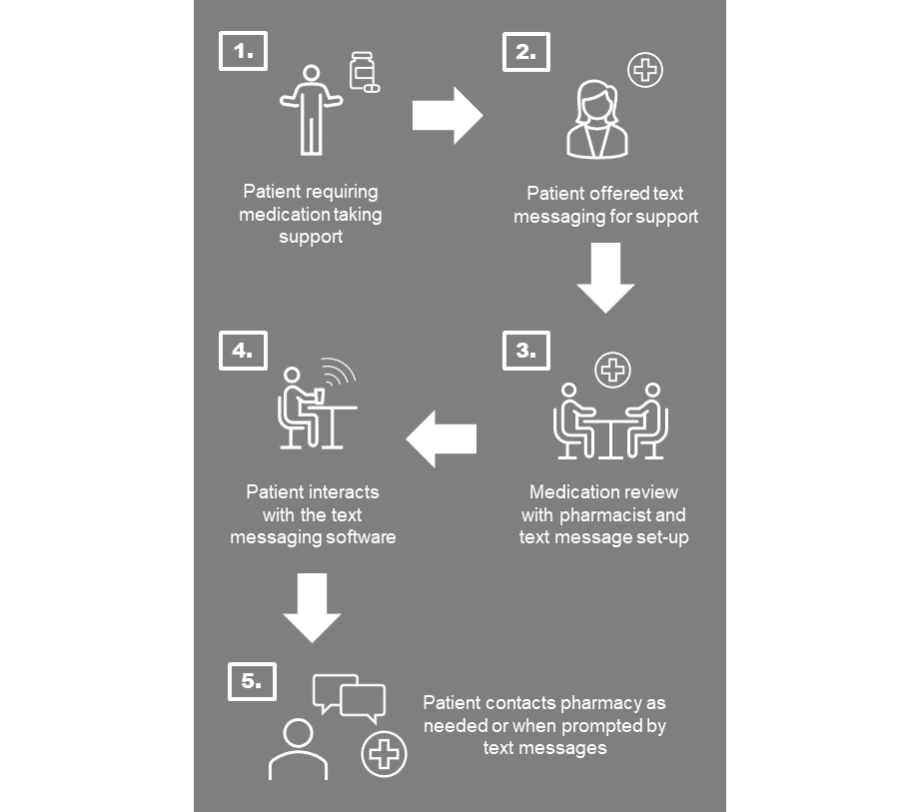
Patient “Journey Map” for the proposed intervention combining automated 2-way text messaging and community pharmacy support.

Using the “journey map” tool from the IDEO.org HCD toolkit, a patient journey for the intervention idea was created (Figure 2) along with a series of design questions which were prioritized with input from the project steering committee. The steering committee included researchers, patients, a general practitioner (GP), community pharmacist, and a representative from the software provider (Florence [24]) to advise on how the technology could support intervention design. To explore the identified design questions, prototypes representing ideas related to the design of the intervention were created. These prototypes were used to gather feedback from the patients and HCPs. A list of design questions, prototypes, and participant groups is presented in Table 1. Who was asked to provide feedback on what prototype depended on the design questions to be answered. At this stage in the design process, we only sought feedback on text message content ideas from HCPs to obtain feedback on clinical acceptability, with the intention of receiving feedback on text message content from patients at a future stage in the development process. This was to ensure that we did not spend time creating text message content, which HCPs would not support the delivery of.

Six prototypes were created to support the discussion of the intervention design concept with the patients and HCPs. These included the following: (1) a video of a community pharmacy assistant inviting a patient to receive the new intervention; (2) a questionnaire to tailor the content of the automated 2-way text messaging; (3) an information leaflet for patients; (4) a video of the pharmacist consultation adapted to deliver the new intervention; (5) a document describing how the tailoring questionnaire would determine text messaging content; and (6) a diagram suggesting how community pharmacy and general practice teams might collaborate to support patients during intervention delivery.

A summary of how each prototype was developed can be found in Multimedia Appendix 2 [6,25-33]. The prototypes represented ideas about both intervention and implementation. As good acceptability is known to be important for intervention implementation [34], we wanted to explore this with key stakeholders, in addition to identifying potential changes to the design. Our key stakeholders included patients as end users and community pharmacists as intervention providers. In addition, we included general practice as the care provider responsible for prescribing and monitoring the medication. The involvement of wider primary care in community pharmacy intervention design has also been highlighted as important by others [35,36].

**Table 1:** Overview of the design questions, prototypes and participant feedback groups for the development study.

Step	Design question	Agreed prioritization with steering committee and rationale	Prototype used to explore	Feedback participant group
2.	What is the best way to approach patients to receive intervention in a community pharmacy setting?	High: as this is a new setting for a digital communication intervention in the United Kingdom	Video of PA^a^ inviting patient to receive the intervention	Patients
2.	Who is the best person to approach them?	Medium: need to check if this needs to be a pharmacist or could be support staff	Video of PA inviting patient to receive the intervention	Patients
2.	What would encourage patients to find out more about the intervention?	High: need to ask about information needs to help patients decide if intervention will be helpful for them	Video of PA inviting patient to receive the intervention	Patients
3.	How should the pharmacist consultation be structured?	High: explore content and delivery for initial acceptability	Video of pharmacist consultation	Patients and HCPs^b^
3.	How would barriers to medication adherence be assessed?	High: explore useability of the tailoring tool, ease of completion	Tailoring questionnaire	Patients
3.	What information will patient need before setting up the text messaging?	High: identify key questions patients have about the text messaging	PIL^c^ for intervention	Patients
3.	What should the information for patients look like?	Medium: explore length and presentation of the information materials	PIL for intervention	Patients
4.	What information should text messages contain?	Medium: information will be taken from other studies but will require a sense check	Document describing the TM^d^ content tailoring process	HCPs
4.	Which messages should we ask patients to respond to?	Medium: information will be taken from other studies but will require a sense check	Document describing the TM content tailoring process	HCPs
4.	What information will we ask patients to send back?	Medium: information will be taken from other studies but will require a sense check	Document describing the TM content tailoring process	HCPs
5.	What happens if the pharmacy needs to refer the patient to another health care professional?	High: need to explore how communication between community pharmacies and general practice might work	Communication diagram	HCPs

^a^PA: pharmacy assistant.

^b^HCP: health care professional.

^c^PIL: patient information leaflet.

^d^TM: text message.

### Feedback Using Modified NGT

#### Overview

Five nominal group meetings based on a modified NGT [23] were arranged to gather and analyze the feedback gathered using the intervention concept prototypes. The participants were provided with prompts to generate ideas about what they liked about the prototype and suggestions for changes. Ideas were initially generated silently, followed by sharing and discussions. Following qualitative analysis, ranking statements were generated and a web-based questionnaire was administered to rank these statements. Only 1 round of ranking was performed.

#### Participants

The participants included patients, community pharmacists, GPs, and practice nurses. Inclusion criteria for patients included active use of a mobile phone and ability to self-manage at least one long-term condition. As this was formative research, we did not wish to initially limit our sampling frame by dictating that participants had more than one long-term condition and felt that those with only one condition would still provide useful feedback at this stage in the development process. HCPs were required to be currently providing patient-facing care.

Convenience sampling was used in this study. Patient participants were recruited through a patient, public, and caregiver involvement network hosted by the University of Sunderland. This network is a collection of people involved in health care teaching and research. HCPs were recruited via professional networks of the research team. Each participant was provided with an invitation letter, participant information sheet, and consent form in advance and were completed before data collection.

#### Ranking Statement Generation

Ranking statements were generated based on a qualitative analysis of the data generated during face-to-face nominal group meetings. The prototypes were presented individually. Elements that participants liked about the prototypes, and what they thought needed to be changed, were captured in the verbal discussion and on participants’ written notes. Topic guides for these discussions are available in Multimedia Appendix 3. The nominal group meetings were audio recorded and transcribed verbatim before qualitative analysis. The meetings were facilitated by GD with contemporaneous notes taken by NH. Meetings were arranged separately for patients and HCPs, as shown in Table 1. Five nominal group meetings were conducted in autumn 2018.

Transcripts and notes were analyzed using the framework approach [23] to identify the statements for the ranking exercise. Initially, an analytic framework was deductively applied. Data were coded for the prototype to which they related, and whether the data were related to a suggested change or aspect that participants liked. Within these categories, individual suggestions were coded inductively to generate ranking statements. Examples of these processes are presented in Table 2. Although the prototypes differed for patients and HCPs, there was some overlapping content. For example, the tailoring questions in the patient prototype also appeared in the HCP prototype which described how the tailoring questionnaire would determine text message content. Where feedback was for a different prototype to that examined directly, we decided to include it in the ranking exercise for the relevant participant group. The analysis was performed using NVivo 11 (QSR International) [37].

**Table 2:** Examples of how ranking statements were generated from qualitative analysis coding.

Qualitative extract from nominal group meetings	Deductive framework application	Inductively coded ranking statement
“I think it was explained well by the pharmacist about what was going to be involved in the scheme” [Patient, Focus Group] 2 “I think [the pharmacy assistant] gave [the patient] plenty of information” [Patient, Focus Group 3]	Prototype: video of pharmacy assistant inviting patient to receive the intervention like statement	Right information given to allow the patient to make a decision
“…because of the way I’ve answered the questionnaire I won’t get a reminder.” [Patient, Focus Group 3] “A lot of people forget their statins at night. It wasn’t checked that he was taking his statin at night, but if he was forgetting at night, he might want to have it at night to remind him to take his statin” [General practitioner, Focus Group 5]	Prototype: tailoring questionnaire suggested change	Ask whether medication reminders is something the patient would benefit from
“I really liked the MUR… I think having that conversation at the start is really good” [Pharmacist, Focus Group 1] “I think if the pharmacist looked at it and thought hang on a minute, why are they taking that in the morning and it’s definitely something they should be taking at night. It raises maybe a bit more care in the review” [Patient, Focus Group 2]	Prototype: pharmacist consultation video like statement	Including a medication review as part of the set-up
“[The text messages put] the buck on them in a way that they’re going to have to be more responsible and I quite like that.” [Practice Nurse, Focus Group 4] “Minimal impact on clinician burden (patient ownership and responsibility placed on them)” [Extract from notes, Pharmacist, Focus Group 1]	Prototype: document describing how tailoring questionnaire determines text messaging content like statement	The patient self-care emphasis which encourages patients to take responsibility
“So your target can be slightly different. Also depending which medications they’re on, because sometimes you just have to accept that level.” [Practice Nurse, Focus Group 4]	Prototype: diagram suggesting community pharmacy and general practice collaboration suggested change	Confirm individual monitoring targets for patients with GP^a^ practice before using home monitoring (eg, blood pressure targets for patients using home blood pressure monitoring)

^a^GP: general practitioner.

#### Statement Ranking

The ranking statements were transferred to a web-based Qualtrics [38] questionnaire. Two versions of the questionnaire were created, one for patient participants and another for HCP participants, reflecting the prototypes examined in the nominal group meetings. Copies of these are available in Multimedia Appendix 4. Participants were asked to rank 5 statements relating to the elements that they liked and then the suggested change statements for each prototype individually. A ranking questionnaire was sent to all participants at nominal group meetings. The rank for the selected statements was then converted into a weighted score, with statements ranked first given a score of 5, second a score of 4, and so on. These scores were then summed to create a score across all the participants who provided feedback on the prototype.

Using the scores from the NGT ranking, decisions were then made about changes to the intervention design. Changes were made when statements were either ranked in the top 3 most important changes or if the suggested changes were small amendments to documents or processes requiring minimal resource change to the overall intervention.

### Ethics Approval

This study was approved by the London Riverside Research Ethics Committee (reference 18/LO/1201), Health Research Authority (IRAS ID:238,875), and University of Sunderland (reference number 002718). The participants were provided with a £20 (US $24.58) gift voucher. No incentives were provided to HCP participants.

## Results

### Overview

A total of 9 patients participated in 2 meetings, and all but 1 had MLTCs. There were 21 HCPs across the 3 meetings. HCPs included pharmacists (n=7), practice nurses (n=5), and GPs (n=9). The average length of the nominal group meeting was 1 hour 21 minutes, with the fifth meeting intentionally shorter (59 minutes) to replace a meeting at a general practice site. The response rate to the ranking questionnaire was 57% (17/30; 6 patients and 11 HCPs). This means that the ranking score had a theoretical maximum of 30 for an individual statement evaluated by patients, 55 for HCPs, and 85 for statements ranked by both patients and HCPs. For most prototypes, there were more than 5 statements to rank for each prototype; therefore, the minimum ranking score could be 0, where it was not ranked as important by any participant.

The following results are organised by prototype. The change statements from the analysis are provided alongside their rank scores and whether changes were made by us to the intervention following feedback. Across all prototypes there were 51 proposed changes to the intervention generated by the analysis. Of these 51 changes, 27 (53%) were made at this point in the design process.

### Video of a Community Pharmacy Assistant Inviting a Patient to Receive the New Intervention (Patient Feedback Only)

This video prototype showed a pharmacy assistant offering the new intervention to a patient waiting to collect their prescription from a community pharmacy. Following an initial expression of interest, the video showed that the patient was provided with the tailoring questionnaire and asked to complete it before a consultation with the pharmacist. The NGT statements for the aspects participants liked about this idea and be found in Table 3, and the suggested changes can be found in Table 4.

Feedback from the patient participants about the proposed design was generally positive. Patients liked the informal approach shown, and most felt that there was enough information provided for patients to decide whether they wanted to find out more. Patients also liked that there was no requirement for the patient to be identified as nonadherent to their medicines, and there was no pressure placed on the patient to sign up.

Most of the suggested changes were incorporated into a reiterated design; however, as the invitation to receive the intervention specified that it involved text messaging, we felt that actively asking patients if they had a mobile phone was an unnecessary change. The suggested change to offer the intervention to patients when problems were identified in a medication review was sensible, but as the intervention was designed for a scenario with no clinical issues to address, this represented a significant change to the context of the intervention and was therefore beyond the scope of the current intervention.

Overall, we were able to answer our original design questions regarding how patients might be invited to receive the proposed intervention. The use of a pharmacy assistant was not raised as something to change, and patients liked that the invitation used the preexisting relationship between the patient and pharmacy assistant. Using wording, which was nonjudgmental and focused on the intervention, increasing motivation to take medicines, was seen as a good way to encourage patients to find out more.

**Table 3:** Summary of Nominal Group Technique statements and scores for aspects that the participants liked about the video of pharmacy assistant inviting patients to the intervention.

Like statements	Ranking score (maximum 30)	Retained in design?
Right information given to allow the patient to make a decision	17	Yes
The informal approach	16	Yes
No pressure was put on the patient to sign up	15	Yes
The introduction was very general, not targeted at a specific patient based on a judgment of their previous compliance	13	Yes
There was an open amount of time given to complete the questionnaire	12	Yes
That it was built on an existing relationship between the patient and the pharmacy assistant	11	Yes

**Table 4:** Summary of Nominal Group Technique statements and scores for suggested changes to the video of pharmacy assistant inviting patients to the intervention.

Change statements	Ranking score (maximum 30)	Design changed?
Patient should be offered help to complete the questionnaire if they need it	28	Yes
The patient information leaflet should be offered before the patient is asked to complete the questionnaire	19	Yes
Patient should be offered the option to complete the questionnaire in the consultation room or at home and bring in later	16	Yes
Communication should be at the same level (eg, both sitting down or both standing)	15	Yes
The pharmacy assistant should ask the patient if they have a mobile phone before introducing them to the service	13	No
There needs to be a way of offering the service to patients who may have medicines delivered or who are housebound	11	Yes
Pharmacists should also offer the service if issues are identified as part of a medication review	5	No

### Tailoring Questionnaire (Patient Feedback Only)

The tailored questionnaire prototype was designed to assess patient suitability for the proposed intervention and to inform the selection of text message content. This included an assessment of medication perceptions using the Beliefs about Medicines Questionnaire [25], the Automaticity subscale of the Self-Reported Habit Index [39], and questions about perceived medicine effectiveness adapted from Phillips et al [26] (see Multimedia Appendix 2 for further details). Patient participants were asked to complete the questionnaire before providing feedback. The feedback statements and ranking scores are presented in Tables 5 and 6.

Patients felt that the questionnaire was clear and easy to complete. During the meeting discussion, patients requested more information about how the questionnaire responses would be used to select the text message content. Further information was provided based on the suggestions in the prototype for principles of intervention tailoring. After receiving this information, patients felt they should be able to choose reminder text messages rather than this being decided by an algorithm, and this was then the highest-ranked statement for change.

Another suggested change was to remove the “neither agree nor disagree” option from the responses. However, as these responses are components of validated tools, their removal was not felt to be appropriate. Participants reported that a question about caregivers would be helpful in understanding medication use. However, as carers would be a different end user group, this was beyond the scope of our intervention.

As the proposed intervention was designed for delivery in MLTCs, whether the questionnaire felt appropriate for patients in this context was a key question to answer using this prototype. Although it did not receive a high score in the ranking exercise, the feedback provided reassurance that the tailoring questionnaire was suitable for use in this group. One key change suggested by participants relating to MLTCs was to discuss long-term conditions verbally with the pharmacist and for pharmacists to complete this section of the questionnaire liaising with the patients’ GPs where needed, instead of patients completing this section using tick-boxes as suggested in the questionnaire prototype.

**Table 5:** Summary of Nominal Group Technique statements and scores for aspects that participants liked about the intervention tailoring questionnaire.

Like statements	Ranking score (maximum 30)	Retained in design?
Easy to read and understand	22	Yes
Clear layout	22	Yes
Use of tick-boxes for most of the questions	21	Yes
Questions did not feel too intrusive	17	Yes
Felt that my responses would identify any problems to address	2	Yes

**Table 6:** Summary of Nominal Group Technique statements and scores for suggested changes to the intervention tailoring questionnaire.

Change statements	Ranking score (maximum 30)	Design changed?
Ask whether medication reminders is something the patient would benefit from	18	Yes
Remove “neither agree nor disagree” option in the questionnaire responses so that people have to answer positively or negatively	15	No
Add in a space for the phone number to be given	14	Yes
Add a question asking if the patient has regular carers	12	No
Pharmacist completes long-term conditions, liaising with the GP^a^ surgery instead of the patient completing this on the form	11	Yes
Add an additional statement in the questionnaire about medicines taking routine (eg, I have a routine for taking my medicines)	11	No
Add in a question to ask about who looks after the phone contract (eg, son or daughter)	9	Yes
Ask whether people would like information about text to voice functions available on their phone	0	No

^a^GP: general practitioner.

### Patient Information Leaflet (Patient Feedback Only)

The patient information leaflet aimed to provide information to prospective users of the intervention and support interaction with automated text messaging. Feedback from the NGT exercise revealed that the leaflet was easy to read and understand. The highest-ranked suggested changes included adding more information on what to expect during text messaging, such as the time for the system to respond and what would happen if patients made a typographical error when replying to text messages. The statements and their ranks are listed in Tables 7 and 8.

This feedback successfully answered the design questions regarding patients’ information needs. Issues for delivering an intervention for MLTCs were raised again with this prototype, as participants requested more examples of text message content beyond the 4 examples included. However, it is unlikely that a full spectrum of examples would be feasible to include, so we chose not to make this change, but this would be subject to further testing using the experience from a future design phase.

**Table 7:** Summary of Nominal Group Technique statements and scores for the aspects participants liked about the intervention patient information leaflet.

Like statements	Ranking score (maximum 30)	Retained in design?
Easy to read and understand	29	Yes
Clear layout	21	Yes
Real examples of text messages the patient might receive	18	Yes
Covered most of the information the patient would need	14	Yes
Comments from other people who have used the service	8	Yes

**Table 8:** Summary of Nominal Group Technique statements and scores for suggested changes to the intervention patient information leaflet.

Change statements	Ranking score (maximum 30)	Design changed?
Add information on how long it will take Flo to respond	27	Yes
Include information on what happens if patient uses an error (eg, typo) in the message	26	Yes
Use real photos rather than graphics (eg, ClipArt)	23	Yes
Add space for a pharmacy stamp with name and contact details	20	Yes
Add in information about NHS 111	19	No
Include more general message examples (eg, not specific to high blood pressure)	17	No
Make emergency information more prominent	15	Yes
Change references to “SMS” to “text message”	6	Yes
Change “Flo says hello” to something more formal	0	No

### Video of Pharmacist Consultation (Patient and HCP Feedback)

The video of the pharmacist consultation included the pharmacist reviewing the tailoring questionnaire and adding the patient to the software system, including receipt and sending of a confirmation text message by the patient. A summary of the aspects that participants liked is presented in Table 9 with the suggested changes in Table 10. As feedback was gathered on this prototype from both patients and HCPs, scores were presented for both groups along with the total score.

There was positive feedback from most participants on the pharmacist consultation structure proposed in the video prototype. Participants liked the Medicine Use Review format and felt that the medication review provided an opportunity to address medication-related issues that could not be addressed by text messaging. The text messaging set-up was also felt to work well, with participants liking a clear explanation of the service being offered and that this included personalizing the times that text messages were sent. Using face-to-face consultation was also highly valued by the patients and HCP participants.

Adding in a written consent process as part of the consultation was the highest-ranked change by HCPs, mainly due to high ranking by community pharmacists. Written consent was prevalent in this setting at the time of data collection. However, these processes have since been removed, in part because of the COVID-19 pandemic; therefore, these processes have not been added for future implementation. Adding verbal information about data protection was a suggested change by HCPs but was a low priority for patients and therefore not prioritized for change in the next stage of intervention design as part of the consultation. This may be because information about this was provided in the patient information leaflet which was reviewed by patients, but not HCPs.

Patient participants’ highest-ranked change was to include a verbal explanation that *Flo*, the persona used for the text messaging interaction, was not a real person. This information was provided in the patient information leaflet, but the patient participants felt that it was sufficiently important that it should also appear in the pharmacist consultation. Patient participants also suggested ensuring that long-term conditions and medication timing were captured and checked as part of the consultation rather than in the tailoring questionnaire. This was suggested so that any clinical issues could be identified, and support the selection of timing for text message reminders. These changes were included in the future iterations of the design. This prototype was successful in exploring our design questions regarding consultation content and delivery for our proposed intervention.

**Table 9:** Summary of Nominal Group Technique statements and scores for aspects participants liked about the pharmacist consultation video.

Like statements	HCP^a^ rank score (maximum 55)	Patients rank score (maximum 30)	Total rank score (maximum 85)	Retained in design?
A clear explanation of the service being offered	36	17	53	Yes
Using a face-to-face method of communication	25	19	44	Yes
Ability of patients to choose the times messages were sent	22	5	27	Yes
Including a medication review as part of the set-up	18	9	27	Yes
Checking if the patient is experiencing any side effects from medication	10	9	19	Yes
Clear communication that the patient can opt out of receiving messages at any time	7	8	15	Yes
Providing a patient information leaflet	7	6	13	Yes
The opportunity to address adherence problems not covered by text messages	10	2	12	Yes
The use of Flo as a persona to communicate with	9	3	12	Yes
Taking place in a private consultation room	7	5	12	Yes
Explanation about the costs of participating to the patient	1	7	8	Yes
Setting up the service with a message in the consultation	7	0	7	Yes
Use of home monitoring equipment and sending in readings	6	0	6	Yes

^a^HCP: health care professional.

**Table 10:** Summary of Nominal Group Technique statements and scores for suggested changes to intervention pharmacist consultation based on video prototype.

Change statements	HCP^a^ rank score (maximum 55)	Patients rank score (maximum 30)	Total rank score (maximum 85)	Design changed?
Add in a more formal written consent process (eg, sign a consent form)	24	7	31	No
Make sure that timing of medication taking is captured and checked	17	14	31	Yes
Check patient knows how to correctly use home monitoring equipment in the consultation before use (eg, peak flowmeter)	22	7	29	Yes
Include a verbal explanation that Flo is not a real person	13	15	28	Yes
Cover data protection and regulation in the verbal consent process	18	6	24	No
Talk about the expected benefits of using text messages to support medicines taking	12	8	20	No
Option for consultation to be done in patients’ home	11	9	20	No
Ensure that home blood pressure monitoring equipment is accurate (calibrated) prior to use	13	4	17	No
Confirm long-term conditions as part of the consultation	2	13	15	Yes
Add a question to assess adherence (eg, how many doses have you missed in the last 7 days)	9	4	13	No
Add in verbal instructions on how to cancel text messages	5	3	8	No
Provide an estimation of how many text messages the patient is likely to receive	8	0	8	No

^a^HCP: health care professional.

### Text Message Content Tailoring Document (HCP Feedback Only)

The feedback from HCP participants on the document describing how text message content would be tailored can be found in Tables 11 and 12. The suggested tailoring process and the proposed text message content were well received, with participants liking the emphasis on self-care. Participants felt that the inclusion of medication reminders was a valuable component and liked that the suggested feedback and monitoring of medication taking allowed for “imperfect” adherence.

However, some of the example text messages were found to be inappropriate for some patients, especially those linked to the more extreme consequences of uncontrolled diseases. This included messages about the risk of amputation and excess health care costs associated with uncontrolled diabetes. It was agreed that these messages would be best maintained for future testing with patients.

A suggestion to provide home monitoring devices was also not changed, as this was felt to be unrealistic in the NHS given the potentially large number of patients who could receive the intervention. Another suggestion was to add a question about routine medication use, which would be unvalidated; therefore, it was also not included in the next design phase iteration. Using the intervention to support side effect monitoring was also suggested; however, we felt that the proposed pharmacist consultation was a better method to assess this.

Our design questions about what information should be requested from patients by the text messaging intervention were only partially answered by this feedback. The participants indicated that they liked the 2-way interaction, but there was little detail in the feedback on specific examples. However, there were no suggested changes in the feedback on the examples provided in the prototype. This suggests that the examples in the prototype were acceptable; however, this requires further investigation.

**Table 11:** Summary of Nominal Group Technique statements and scores for aspects participants liked about the document showing text message content selection based on tailoring questionnaire.

Like statements	Ranking score (maximum 55)	Retained in design?
The patient self-care emphasis which encourages patients to take responsibility	33	Yes
The tailoring of content to individual patients	30	Yes
Realistic targets which allow “imperfect” adherence	20	Yes
Providing information in smaller “chunks” which may be easier for the patient to digest	17	Yes
The simple language used in the messages	16	Yes
The inclusion of prompts or cues to support medicines taking	14	Yes
Messages tailored to patients’ beliefs about medication	14	Yes
Messages encouraging patients to get feedback on medicines taking (eg, blood pressure)	8	Yes
Two-way communication between the patient and Flo	7	Yes
Prioritization of concerns, then necessity, then experience, then habit	4	Yes
That the intervention is automated	1	Yes
The use of habit as a model for the messages	1	Yes

**Table 12:** Summary of Nominal Group Technique statements and scores for suggested changes to the document showing text message content selection based on tailoring questionnaire.

Change statements	Ranking score (maximum 55)	Design changed?
Create layers of messages, with more dramatic messages (eg, amputation being reserved for those with persistent nonadherence)	30	No
Reword the behavior experimentation message to seek approval from a health care professional before stopping medication to notice any impact	29	Yes
Provide home monitoring devices (eg, blood pressure monitor where messages are indicated but patients do not have the equipment)	23	No
Add an additional statement in the questionnaire about medicines taking routine (eg, I have a routine for taking my medicines)	22	No
Remove “neither agree nor disagree” option in the questionnaire responses so that people have to answer positively or negatively	21	No
Add in side effect monitoring as part of the intervention	17	No
Remove requirement to input keywords in responses such as “MEDS” or “DAYS”	10	No

### Community Pharmacy and General Practice Collaboration Diagram (HCP Feedback Only)

The prototype showing how community pharmacy and general practice teams might work together to support patients receiving the new intervention is outlined in a document showing a series of flow diagrams. The proposed text messaging software allows all HCPs to access the same patient data (with patient permission), which has been highlighted as a method to support communication. Feedback from participants in the suggested collaboration model is provided in Tables 13 and 14. Participants liked the community pharmacy-led design of the proposed intervention, and the data were still accessible to all HCPs through the software provider. Community pharmacist participants also liked the proposed use of pharmacy support staff to distribute the workload associated with the intervention.

The highest-ranked suggested change was to confirm patients’ individual monitoring targets (such as blood pressure) with general practices. However, this could add significantly to the intervention set-up. In addition, as blood pressure targets are standardized in clinical guidance, we felt that the intervention should reflect these targets. However, this should be explored with patients using the system in the next stage of design development.

Our design question to answer using this prototype was to explore how pharmacies would coordinate with general practices when patients required clinical input or changes to medicines. Participants provided useful insights and suggestions on how best to achieve this based on current health care processes. This included a suggestion for general practice to add notifications indicating patient participation in the intervention into patient records. The participants also suggested that pharmacists should coordinate patient support arising from the intervention rather than patients directly contacting their general practice, as indicated in the prototype. This approach reflects other community pharmacy-led interventions such as the New Medicines Service.

There was some uncertainty in the feedback on how much information general practice staff needed about the intervention. The suggestion of using a website to provide more detailed information is desirable. However, the suggested changes included both that practices only needed to be informed as courtesy and that notifications should include more detail. These would seem to be opposing pieces of feedback and, therefore, further exploration is needed about what level of information general practices might want and at what point.

The link between the intervention and the delivery of regular care by community pharmacies was also highlighted in the feedback on this prototype. There was a suggested change in that only a patient’s nominated pharmacy could provide the intervention. The nominated pharmacy automatically receives patient prescriptions from the NHS Electronic Prescription Service. This information is easily visible in general practice and is therefore seen as an important prerequisite for patients receiving the intervention. Pharmacies also felt that follow-up with patients shortly after initiating the intervention would be helpful to check that patients seemed to be receiving the correct text messages and any initial issues could be addressed. Participants also highlighted the need to ensure that patients could access support when they received a text message telling them to contact an HCP. This led to a change in the intervention to ensure that text messages are only sent to patients from Monday to Thursday.

**Table 13:** Summary of Nominal Group Technique statements and scores for aspects participants liked about the suggested collaboration model in community pharmacies and general practices.

Like statements	Ranking score (maximum 55)	Retained in design?
Community pharmacy-led service	41	Yes
That data is accessible to all health care professionals	40	Yes
Process is clear and makes sense	33	Yes
Makes good use of pharmacy support staff	20	Yes
Use of PharmOutcomes (a software platform for community pharmacy teams)	16	Yes
A website can act as a portal for more detailed information about specific content where needed	15	Yes

**Table 14:** Summary of Nominal Group Technique statements and scores for suggested changes to the collaboration model in community pharmacies and general practices.

Change statements	Ranking score (maximum 55)	Design changed?
Confirm individual monitoring targets for patients with GP^a^ practice prior to using home monitoring (eg, blood pressure targets for patients using home blood pressure monitoring)	28	No
GP practices should add notification of patient using Flo to GP record, to ensure any medication changes are communicated to the pharmacy	23	Yes
Community pharmacies should contact the GP practice on behalf of patients initially where queries arise	22	Yes
Notification to practices should include which protocols have been set up for patients	20	Yes
General practice should receive notification of set up for information only	17	Yes
Add in a message to ask if the patient is happy with the messages so far shortly after initiation of intervention	17	Yes
The nominated pharmacy should be the only one able to provide the service	16	Yes
Messages should only be sent Monday to Thursday to allow quick access to health care professionals where there are queries	13	Yes

^a^GP: general practitioner.

## Discussion

### Principal Findings

This paper includes a detailed description of our intervention, methods, and HCD approach used in its design and development. It provides a novel worked example of the application of HCD in developing prototypes for a complex health care intervention. Our HCD approach combined with modified NGT allowed us to successfully obtain and prioritize feedback from key stakeholders on our intervention concept for a community pharmacy–delivered text messaging intervention to support medication taking. The process facilitated the development of our proposed intervention and informed changes to the intervention materials, including the tailoring questionnaire, patient information leaflet, and pharmacist consultation. The proposed implementation of the intervention was found to be acceptable to patients, community pharmacists, and general practice staff. Suggestions for changes in how the intervention will be implemented will be carried forward in the next iteration of the design.

Our findings provide confidence that our research-informed intervention will be acceptable and implementable to all key stakeholders. The open nature of the feedback gathering, aligned with the principles of HCD, led to some suggestions in the data that were either outside the current design scope or contradicted the evidence base used to create the initial prototypes. In these cases, we labeled these as changes that were not implemented, although feedback could inform future alternative designs. The experience of the steering group was also used to make design change decisions alongside feedback from participants in this study, which also led to some suggestions not being taken forward. However, the generation and ranking of the feedback from key stakeholders in this study ensured that these opinions were heard and considered equally as part of the design process and reflected the intention of a co-design process similar to that suggested elsehere [40].

### Utility of HCD and Prototypes for the Proposed Intervention Design

Digital health care interventions do not exist in isolation; they are integrated into existing clinical pathways, so designing and gaining feedback on the whole patient journey as per HCD principles is important. For example, the discussions in our nominal group meetings were not always limited to one prototype at a time. Participants reflected on the relationship between the prototypes as the discussion progressed, leading to new feedback on the previously discussed prototypes. These ideas would not have been captured if feedback had been collected on individual components of the intervention at separate times. Our approach also allowed participants to see the relationships between the digital and nondigital components of the complex intervention, which can be overlooked in digital health care intervention design.

As the number of individuals involved in a co-design process is often limited, there is a potential risk that design outputs can ignore important evidence-based ideas. The process of creating prototypes allowed the incorporation of evidence and a theoretically informed approach at the start of the design process, but with the opportunity for the approach to be “sense checked” by key stakeholders. The unknowns associated with translating the research evidence into a novel setting could also be explicitly explored through our process, such as the acceptability of delivering text messaging from community pharmacies.

By using prototypes of the intervention, we were also able to explore a range of design questions without the need to build and test the intervention in the “real world.” This approach has the potential to minimize research waste, as feedback can be gathered on ideas early in the design process, preventing time spent on undesirable intervention components, or even stopping intervention design altogether where ideas are not acceptable to important stakeholders. Our approach may be particularly helpful when the design questions are more focused because evidence exists for most aspects of the intervention, but smaller changes, such as delivery context (as was the case in this scenario), require exploration.

### Using NGT to Gather Feedback on Proposed Intervention Design

Using NGT to structure data collection and analysis increases the robustness of feedback gathering. Collating feedback statements across meetings for ranking allowed everyone to consider all the generated statements. This was particularly useful for integrating feedback from both patients and HCPs in the pharmacist consultation prototype. Here, the elements that participants liked were ranked similarly, but the priority for changes differed between the participant groups. Overall, the questions we as designers thought were important (as articulated in our design questions) often did not rank highly as important by our participants. This provides evidence for the differing priorities between stakeholders and intervention designers, and the need for multiperspective input into complex health care intervention design.

The prioritization of feedback using NGT also helped us discern which aspects of the intervention and changes participants felt most strongly about. This was useful for suggestions that dominated discussions in meetings, which, if only analyzed qualitatively, may have indicated greater importance than the NGT scores revealed. However, as we did not have a high response rate, this could also be due to participants raising these issues in meetings, but not completing the ranking questionnaire.

We feel that using NGT offers a potential solution to some of the challenges associated with co-design processes, particularly the need to integrate feedback and ideas from a range of perspectives. The NGT also seemed to complement the use of prototypes, as there was consistency in how ideas were communicated to participants and, therefore, confidence that all feedback was linked to the same design ideas.

### Comparison With Development Methods for Similar Interventions

We chose an HCD process as we felt it could accommodate both the digital and nondigital aspects of our proposed intervention and to ensure that we were creating a solution that met patients’ needs around medication taking. Guidance on developing digital-only health care interventions has been published by Abroms et al [40] and has been used for other digital communication interventions currently in development in the United Kingdom to support medication use [41,42]. Abroms et al [40] recommended the use of many of the features presented in this study, including a theoretically informed behavioral approach and the design of a delivery framework.

Other United Kingdom-based digital communication interventions to improve medication adherence have been designed for delivery from the general practice setting [41,42] and have used different approaches to ours. Interventions by Bartlett et al [42] for patients with diabetes and by Kassavou et al [43,44] for patients with hypertension and type 2 diabetes both seemed to focus initially on the digital communication content rather than implementation during the developmental process, which were explored simultaneously in our study.

### Limitations

The discussions within nominal group meetings were not as multidisciplinary as we hoped to achieve. One nominal group meeting contained only pharmacists, and another was predominantly practice nurses. One meeting was multidisciplinary but was based on a single general practice. Combined, the data seem to offer a good range of feedback on the initial design concept for the new intervention but may have benefited from a more multidisciplinary discussion.

Research using this type of methodology can be limited by the self-selection of participants in the studies in which they are interested. Therefore, broader perspectives may have been omitted from our results. Recruiting patient participants from a university-based network within a pharmacy school may have also increased the acceptability of the intervention. Our inclusion criteria also did not specify that patient participants needed to have MLTCs; however, most of our patients did, and many also drew on their experiences as caregivers for those who did. As these findings are just a starting point for further exploration and iteration, we believe that these limitations did not significantly affect the intervention development process.

Our modified version of the NGT included only 1 round of ranking that was not shared to inform a second round of discussion and voting, as per a normal NGT process. This means that we captured the individual perspectives of our participants but may have achieved a stronger consensus if a second round of discussion and ranking was conducted. However, given that we had a low response rate for the initial ranking questionnaire, we suspect that engagement in any further rounds of feedback and ranking would likely be poor. As we also considered all suggestions from the NGT data collection as part of a co-design process, we believe that generating a stronger consensus as defined by larger ranking scores across a smaller number of statements would be unlikely to change the final design decisions we made.

### Further Development and Research

The next step in intervention design is to develop a library of text messages for MLTCs that can be delivered using the approach described here. This will be used in a future study to further explore the delivery model using the IDEO.org “Live Prototyping” process, incorporating the changes from our results. This is important for gathering patient feedback on text message content, which was not included at this stage in the design process. Future studies should design training programs for community pharmacies and communication tools to be used in general practice. Each of these are intended to lead to an evaluation of the intervention in a real-world setting, which will also enable the testing of our intervention program theory.

### Conclusions

Combining HCD with NGT allowed us to create a research-informed design for a text messaging intervention for medication adherence, gather feedback from key stakeholders, and reiterate for future testing. Although HCD has been proposed as a potential strategy for developing complex interventions in recent MRC guidance, examples of using such an approach are lacking. Our work can serve as a model for developing complex health care interventions in the future, especially where they combine digital and nondigital components.

## Appendix

Multimedia Appendix 1 Guidance for reporting intervention development studies in health research checklist.

Multimedia Appendix 2 Detailed descriptions of prototype development.

Multimedia Appendix 3 Nominal group topic guides.

Multimedia Appendix 4 Ranking questionnaires.

## Abbreviations

COM-B: Capability, Opportunity, and Motivation model
GP: general practitioner
HCD: human-centered design
HCP: health care professional
MLTC: multiple long-term condition
MRC: Medical Research Council
NGT: Nominal Group Technique
NHS: National Health Service

## Data Availability

We did not seek consent from participants or approval for data sharing beyond the study team; however, data requests may be made to the corresponding author for consideration on a case-by-case basis.

## References

[1] Organisation mondiale de la santé, World Health Organization (2003). Adherence to Long-term Therapies Evidence for Action.

[2] Cutler RL, Fernandez-Llimos F, Frommer M, Benrimoj C, Garcia-Cardenas V (2018). Economic impact of medication non-adherence by disease groups: a systematic review. BMJ Open.

[3] Barnett K, Mercer SW, Norbury M, Watt G, Wyke S, Guthrie B (2012). Epidemiology of multimorbidity and implications for health care, research, and medical education: a cross-sectional study. Lancet.

[4] Vetrano DL, Bianchini E, Onder G, Cricelli I, Cricelli C, Bernabei R, Bettoncelli G, Lapi F (2017). Poor adherence to chronic obstructive pulmonary disease medications in primary care: role of age, disease burden and polypharmacy. Geriatr Gerontol Int.

[5] Nieuwlaat R, Wilczynski N, Navarro T, Hobson N, Jeffery R, Keepanasseril A, Agoritsas T, Mistry N, Iorio A, Jack S, Sivaramalingam B, Iserman E, Mustafa RA, Jedraszewski D, Cotoi C, Haynes RB (2014). Interventions for enhancing medication adherence. Cochrane Database Syst Rev.

[6] Donovan G, Hall N, Ling J, Smith F, Wilkes S (2022). Influencing medication taking behaviors using automated two-way digital communication: a narrative synthesis systematic review informed by the Behavior Change Wheel. Br J Health Psychol.

[7] O'Cathain A, Croot L, Duncan E, Rousseau N, Sworn K, Turner KM, Yardley L, Hoddinott P (2019). Guidance on how to develop complex interventions to improve health and healthcare. BMJ Open.

[8] Fenerty SD, West C, Davis SA, Kaplan SG, Feldman SR (2012). The effect of reminder systems on patients' adherence to treatment. Patient Prefer Adherence.

[9] Mistry N, Keepanasseril A, Wilczynski NL, Nieuwlaat R, Ravall M, Haynes RB, Patient Adherence Review Team (2015). Technology-mediated interventions for enhancing medication adherence. J Am Med Inform Assoc.

[10] Ciciriello S, Johnston RV, Osborne RH, Wicks I, deKroo T, Clerehan R, O'Neill C, Buchbinder R (2013). Multimedia educational interventions for consumers about prescribed and over-the-counter medications. Cochrane Database Syst Rev.

[11] Registers. General Pharmaceutical Council.

[12] (2021). General pharmaceutical services in England 2015/16 - 2020/21. NHS Business Services Authority.

[13] (2013). New medicine service (NMS). Pharmaceutical Services Negotiating Committee.

[14] Elliott RA, Boyd MJ, Tanajewski L, Barber N, Gkountouras G, Avery AJ, Mehta R, Davies JE, Salema N, Craig C, Latif A, Waring J, Chuter A (2020). 'New Medicine Service': supporting adherence in people starting a new medication for a long-term condition: 26-week follow-up of a pragmatic randomised controlled trial. BMJ Qual Saf.

[15] Latif A, Pollock K, Boardman HF (2011). The contribution of the Medicines Use Review (MUR) consultation to counseling practice in community pharmacies. Patient Educ Couns.

[16] (2013). National Pharmacy Services. Pharmaceutical Services Negotiating Committee.

[17] Michie S, van Stralen MM, West R (2011). The behaviour change wheel: a new method for characterising and designing behaviour change interventions. Implement Sci.

[18] Kok G, Gottlieb NH, Peters GY, Mullen PD, Parcel GS, Ruiter RA, Fernández ME, Markham C, Bartholomew LK (2016). A taxonomy of behaviour change methods: an intervention mapping approach. Health Psychol Rev.

[19] Jackson C, Eliasson L, Barber N, Weinman J (2014). Applying COM-B to medication adherence. Eur Health Psychol.

[20] O'Cathain A, Croot L, Sworn K, Duncan E, Rousseau N, Turner K, Yardley L, Hoddinott P (2019). Taxonomy of approaches to developing interventions to improve health: a systematic methods overview. Pilot Feasibility Stud.

[21] IDEO (Firm) (2015). The Field Guide to Human-centered Design Design Kit.

[22] Duncan E, O'Cathain A, Rousseau N, Croot L, Sworn K, Turner KM, Yardley L, Hoddinott P (2020). Guidance for reporting intervention development studies in health research (GUIDED): an evidence-based consensus study. BMJ Open.

[23] Van de Ven A H, Delbecq AL (1972). The nominal group as a research instrument for exploratory health studies. Am J Public Health.

[24] Florence. Generated Health.

[25] Horne R, Weinman J, Hankins M (1999). The beliefs about medicines questionnaire: the development and evaluation of a new method for assessing the cognitive representation of medication. Psychol Health.

[26] Phillips LA, Cohen J, Burns E, Abrams J, Renninger S (2016). Self-management of chronic illness: the role of 'habit' versus reflective factors in exercise and medication adherence. J Behav Med.

[27] Your Introduction to the Florence Telehealth System. Simple Telehealth Member?s Resources.

[28] Verplanken B, Orbell S (2003). Reflections on past behavior: a self-report index of habit strength. J Appl Social Pyschol.

[29] Gardner B, Abraham C, Lally P, de Bruijn G (2012). Towards parsimony in habit measurement: testing the convergent and predictive validity of an automaticity subscale of the Self-Report Habit Index. Int J Behav Nutr Phys Act.

[30] Horne R (1997). Representations of medication and treatment: advances in theory and measurement. Perceptions of Health & Illness.

[31] Cooper V, Buick D, Horne R, Lambert N, Gellaitry G, Leake H, Fisher M (2002). Perceptions of HAART among gay men who declined a treatment offer: preliminary results from an interview-based study. AIDS Care.

[32] Horne R (2003). Treatment perceptions and self-regulation. The Self-regulation of Health and Illness Behaviour.

[33] PharmOutcomes homepage. PharmOutcomes.

[34] Sekhon M, Cartwright M, Francis JJ (2017). Acceptability of healthcare interventions: an overview of reviews and development of a theoretical framework. BMC Health Serv Res.

[35] Latif A, Waring J, Watmough D, Barber N, Chuter A, Davies J, Salema N, Boyd MJ, Elliott RA (2016). Examination of England's New Medicine Service (NMS) of complex health care interventions in community pharmacy. Res Social Adm Pharm.

[36] Watson M, Silver K, Watkins R (2020). "What counts can't always be measured": a qualitative exploration of general practitioners' conceptualisation of quality for community pharmacy services. BMC Fam Pract.

[37] NVivo homepage. NVivo.

[38] Qualtrics XM. Qualtrics.

[39] Aarts H, Verplanken B, Knippenberg A (1998). Predicting behavior from actions in the past: repeated decision making or a matter of habit?. J Appl Social Pyschol.

[40] Abroms LC, Whittaker R, Free C, Mendel Van Alstyne J, Schindler-Ruwisch JM (2015). Developing and pretesting a text messaging program for health behavior change: recommended steps. JMIR Mhealth Uhealth.

[41] Bermon A, Uribe-Rodríguez AF, Pérez-Rivero PF, Prieto-Merino D, Cáceres Rivera DI, Guio E, Atkins L, Horne R, Murray E, Serrano Díaz NC, Free C, Perel P, Casas JP (2019). Evaluation of the efficacy and safety of text messages targeting adherence to cardiovascular medications in secondary prevention: the txt2heart Colombia randomised controlled trial protocol. BMJ Open.

[42] Bartlett YK, Farmer A, Rea R, French DP (2020). Use of brief messages based on behavior change techniques to encourage medication adherence in people with type 2 diabetes: developmental studies. J Med Internet Res.

[43] Kassavou A, Houghton V, Edwards S, Brimicombe J, Sutton S (2019). Development and piloting of a highly tailored digital intervention to support adherence to antihypertensive medications as an adjunct to primary care consultations. BMJ Open.

[44] Kassavou A, Mirzaei V, Brimicombe J, Edwards S, Massou E, Prevost AT, Griffin S, Sutton S (2020). A highly tailored text and voice messaging intervention to improve medication adherence in patients with either or both hypertension and type 2 diabetes in a UK primary care setting: feasibility randomized controlled trial of clinical effectiveness. J Med Internet Res.

