# Supplementary material for: Two-way Automated Text Messaging Support From Community Pharmacies for Medication Taking in Multiple Long-term Conditions: Human-Centered Design With Nominal Group Technique Development Study
Source: JMIR Form Res. 2022 Dec 21;6(12):e41735. doi: 10.2196/41735 (PMC9813818; doi:10.2196/41735)

##

## Prototype development process for a new community pharmacy text messaging intervention to support medication taking

The six prototypes developed for gathering feedback on the new intervention design concept were grounded in a systematic review of similar interventions [6]. Three prototypes were designed to generate feedback from patients, two prototypes for healthcare professional feedback and one was tested across both groups (see Table 1 in the paper). A detailed description of prototypes developed is provided in this appendix.

### Video of community pharmacy assistant inviting a patient to receive the intervention

A video was created to demonstrate how patients might receive the proposed intervention. The invitation was demonstrated as linked to medication supply to represent an opportunistic approach to recruitment. A script was created using language to reflect an open invitation without any prior assumption of medicines taking performance by the patient. The intervention was framed to support motivation with medicines taking, aligned with the underpinning programme theory for the intervention design. The video was recorded in a simulated pharmacy environment with a volunteer pharmacy student and patient researcher. The same patient was used in both the pharmacy invitation video and pharmacist consultation video to help tell the story of the patient experience of the intervention. A copy of the video is available upon request.

### Patient information leaflet about intervention

A mock-up patient information leaflet was drafted based on text from an existing leaflet from the Simple Telehealth Community [27]. Amendments were made to adapt the content for an intervention focussing on medicines taking and delivered from a community pharmacy. A copy is available in Appendix 1 of this document.

###

### Video of pharmacist consultation

The pharmacist consultation was based on a Medicines Use Review (MUR), and the model for using Simple Telehealth in general practice settings. Additions to the MUR included:

- Registering the patient on the Simple Telehealth system
- Acquiring information required to set up text messaging protocols
- Providing information to support engagement with the text messaging intervention

A script was created, and this was then recorded to create the community pharmacist consultation video. A copy of the video is available upon request.

### Tailoring questionnaire for text messaging content

Studies from the systematic review which used intervention tailoring were evaluated. However, most of these could not be used in the context of multimorbidity. The Beliefs about Medicines Questionnaire (BMQ) [25] was identified during the background literature review as a potential tool to evaluate perception based barriers to medication nonadherence to direct text messaging content and increase reflective motivation towards medicines taking. The BMQ was also felt to be useful for patients with MLTCs. This was used as one component of the tailoring questionnaire.

Habit strength was also found in the background literature review to be an important predictor of medication taking behaviour. The Self-Reported Habit Index (SRHI) [28] has been validated as a measurement of habit strength and has been compared to medication taking behaviours. The automaticity subscale of the SRHI (A-SRHI) had also been found to be the most predictive component of the SRHI for habit strength [29]. This was therefore selected as an additional component into the tailoring questionnaire. Philips et al.[26] had also combined A-SRHI with BMQ and two questions which assess patients’ perceptions of medicine effectiveness to further predict medication nonadherence.

Perceived efficacy of medicines is suggested to be contained within the necessity statements of the BMQ [30] but also a distinct construct within this [31]. To account for this, Phillips et al. [26] in their work created two separate questions about perceived effectiveness of medication in their study using a binary yes/no response for one question and a three point scale for the second question. However as both the BMQ and A-SHRI are both rated on a five-point Likert scale, the question about experience of medicines effectiveness from the work by Phillips et al. [26] was transformed into a Likert scale to match other items. Combining these approaches enabled an assessment of reflective and automatic motivation influences on medicines taking to tailored text message content.

Practical questions were also included to screen patients for appropriateness to receive a text messaging programme. These included questions about mobile phone signal, ability to use text messaging, phone contract type, current long-term conditions, timing of medicine self-administration, availability of home monitoring equipment and permission for pharmacies to also use their information to set up the relevant text messaging content. A copy of the questionnaire is available on request.

### Text message content tailoring process document

To represent how the tailoring questionnaire would determine text message content, a document to represent this was created. The document suggested that patients were stratified dependant on their potential needs. The questionnaire responses were separated into different motivation categories that may be influencing medication taking behaviour. The diagram from the prototype which outlines these categories and the order in which they are resolved can be found in Figure 1.

Patients who indicated higher concerns about their medicines were created as a group for priority. Patients would be placed into this ‘Concerns’ category if they indicated strong agreement with concerns about medicines statements from the BMQ (score of ≥15). Horne has also suggested the mid-point score of a BMQ subscale can be used to categorise patients into opposing ends of the scale [30]. These patients would receive content which aimed to reduce their concerns about medication or encourage them to discuss concerns with the pharmacist. Suggested content for these patients included providing information and reassurance about side effects, reflecting research using the BMQ [32]. This would deliver the BCT *Reduce negative emotions*. Other BCTs included in the prototype for this group included *Commitment*, *Monitoring of emotional consequences*, and *Framing/ reframing*. Encouragement to discuss concerns with their healthcare professional delivered the BCT *Social support (unspecified)*.

The second proposed group of patients were those with low score for perceived need for medicines from the BMQ. Using a mid-scale cut-off point, patients would be placed into the ‘Necessity’ category if they had a score of ≤15. Text messaging content for this group was suggested to deliver the ‘Persuasion’ intervention function, including the BCTs *Information about health consequences*, *Salience of consequences*, *Information about social and environmental consequences* and *Credible source*. These aimed to increase the perceived need for medication by participants.

A third group was identified using the responses from the experiential feedback about medicines questions drawn from the work by Phillips et al.[26] but transformed into statements which could be rated on a 5-point Likert scale. These included:

- I have noticed the positive benefits of taking my medication
- I have experienced solid/ convincing evidence that my medication does what it is supposed to do

Using a mid-point cut off scale similar to the approach with the BMQ items, patients were proposed to be placed in a ‘Medication effectiveness’ category with a score ≤6. Content for this patient category included the ‘Education’ and ‘Persuasion’ intervention functions, BCTs for this group were suggested as *Goal setting (outcome)*, *Review outcome goal(s)*, *Self-monitoring of outcome(s) of behaviour*, *Biofeedback*, *Feedback on outcome(s) of behaviour* and *Behavioural experiments*.

The final groups were drawn from habit strength score from the A-SRHI questions. Although both Phillips et al. [26] and Aarts et al.[28] make reference to the relative importance of ‘high’ and ‘low/weak’ habit strength, they did not relate this back to any score values on the A-SHRI. Judgement of the research team was therefore used to create the following categories:

- ‘Low’ habit strength (score ≤8)
- ‘Medium’ habit strength (score 9-15)
- ‘High’ habit strength (score ≥16)

Those with ‘Low’ habit strength were suggested to receive the BCTs *Prompts/cues*, *Monitoring of behaviour by others without feedback*, *Goal setting (behaviour)*, *Review behaviour goal(s)*, *Discrepancy between current behaviour and goal*, *Feedback on behaviour* and *Social reward* for the behaviour of taking medication.

For those with a ‘Medium’ habit, more emphasis was placed on monitoring BCTs including *Monitoring of behaviour by others without feedback*, *Self-monitoring of behaviour*, *Behavioural practice / rehearsal*, *Habit formation* and *Adding objects to the environment*. In those with ‘High’ habit strength the aim would be to maintain this good habit by using the BCT *Monitoring of behaviour by others without feedback*.

Prioritisation order was created by considering the relative importance of these different aspects on medication adherence. Addressing medication concerns was considered to be highest priority due to the significant potential impact on intentional non-adherence and also that these could reflect potentially inappropriate polypharmacy. Addressing perceived necessity was considered next most important to address, followed by perceived effectiveness and finally habit strength.

### Collaboration diagram

The suggested collaboration diagram described how the proposed intervention would work in a community pharmacy and how wider primary care might respond to queries from patients receiving the intervention. The diagram was based on pharmacy services and the current model of Simple Telehealth use in general practice. A copy is available in Appendix 2 of this document.

The first flow diagram showed the suggested process for patient initiation onto the text messaging programme. It reflects the ‘video of pharmacy assistant inviting patients to the intervention’ prototype, the tailoring questionnaire, and the pharmacist consultation. Following text-message set-up as described in the pharmacist consultation video, the flow diagram suggested technical checks to ensure the right text messaging protocols had been set-up for the patient.

This is followed by suggesting how a pharmacy might respond to a patient query because of receiving a text message. The prototype outlined pharmacists being able to handle most ‘simple’ queries, for example questions about side effects or how medicines should be used. However, where a query from a patient might require changes to medication, the flow diagram indicated that pharmacists should liaise with the wider primary care team. The flow diagram also suggested a review of the allocated text messaging protocols following any queries to ensure that messages were still appropriate for the patient. A software system PharmOutcomes [33] was also suggested as a tool to support record keeping.

The prototype also included suggested pathway for how the wider healthcare team might respond to queries from patients resulting from the intervention text messages. This highlighted that Simple Telehealth can be accessed by all healthcare professionals. The prototype also suggested a website to provide information about the intervention to the wider healthcare team.


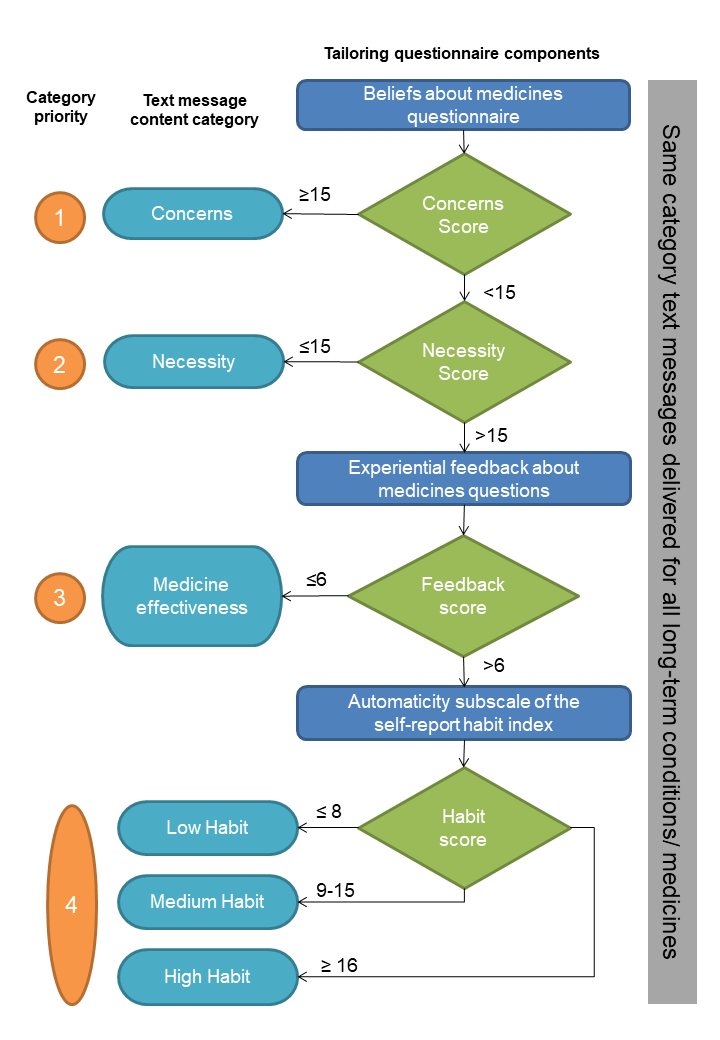


Figure 1 Flow diagram for allocating text message content based on the tailoring questionnaire

Appendix 1 Patient information leaflet prototype


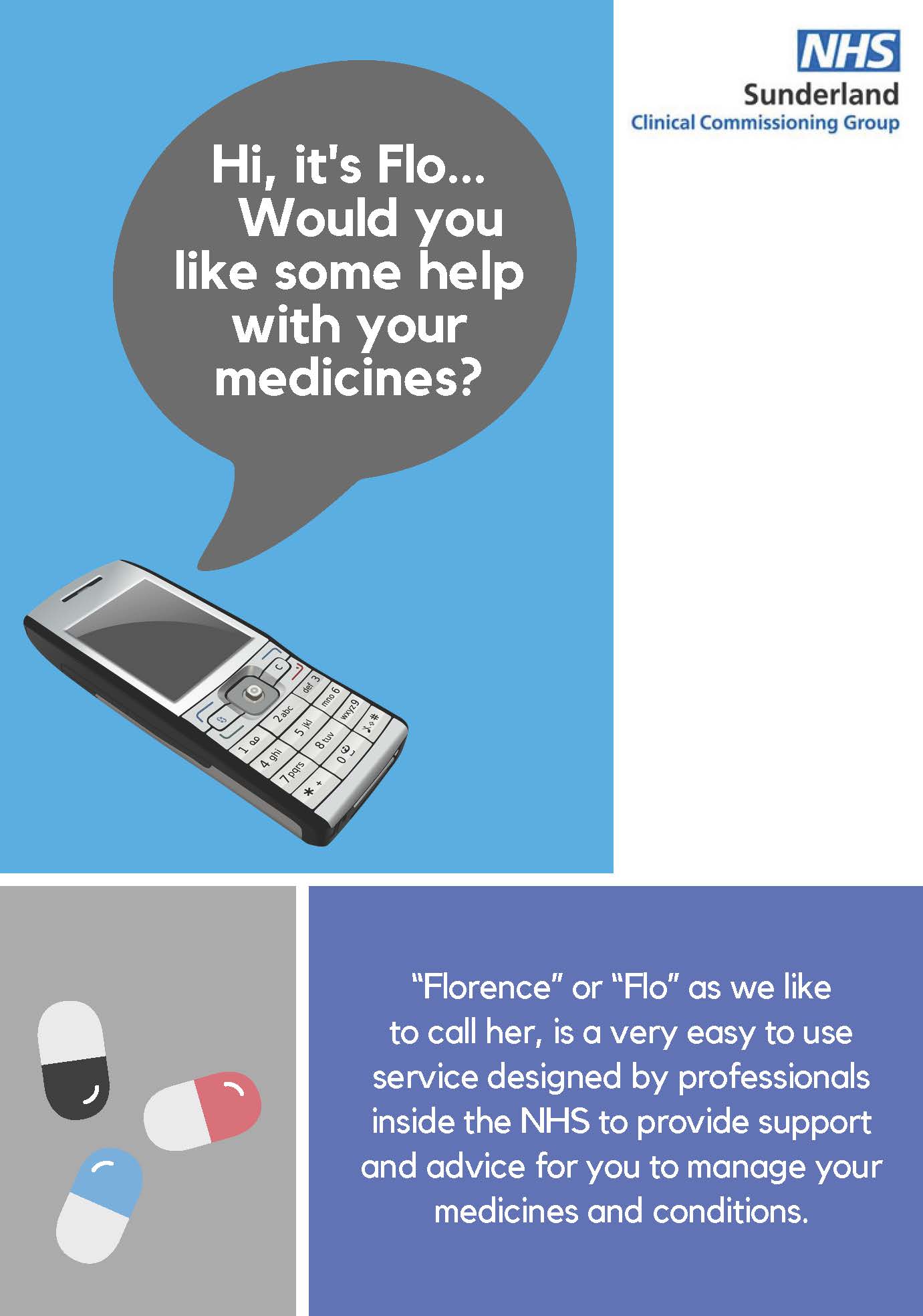

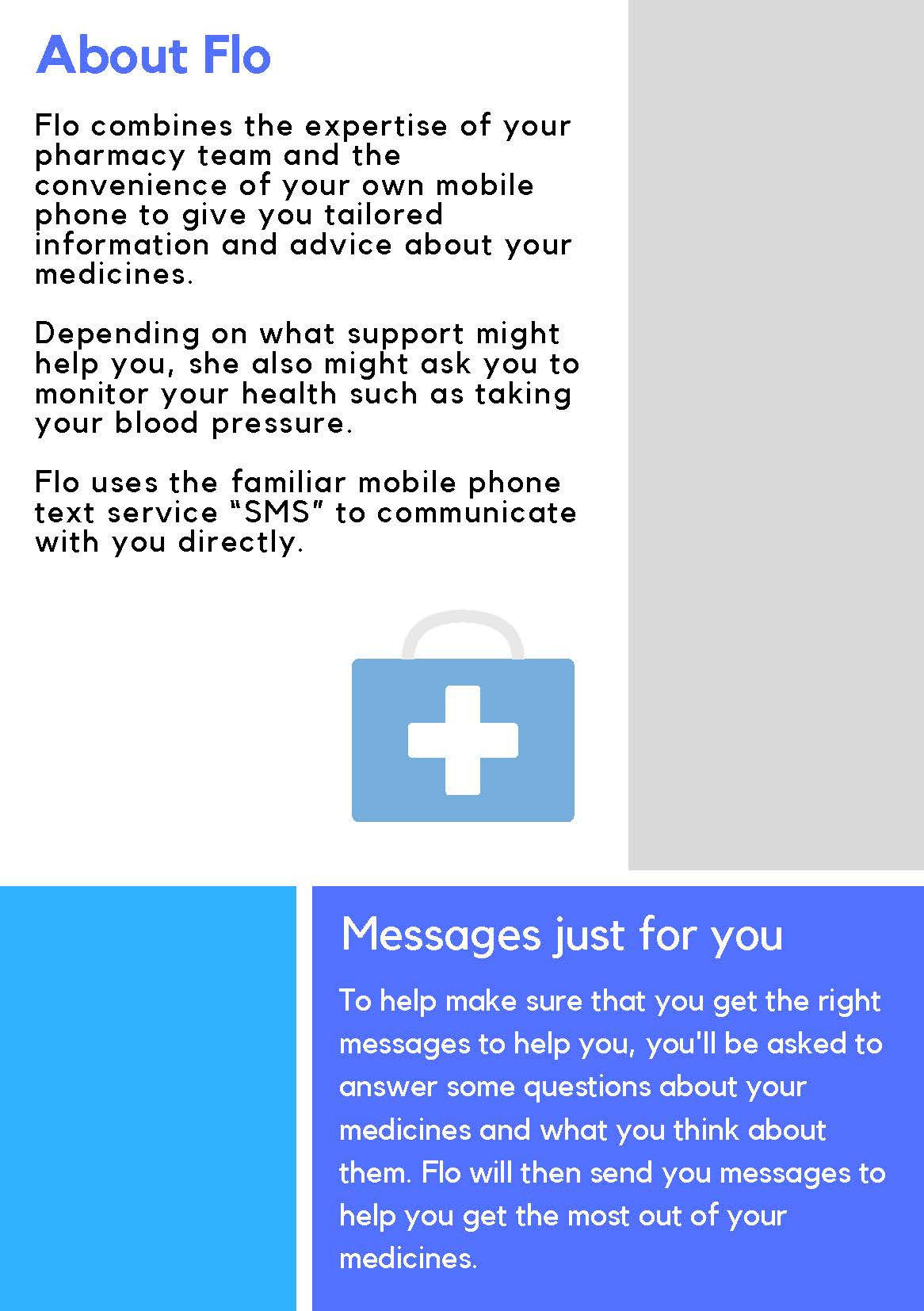

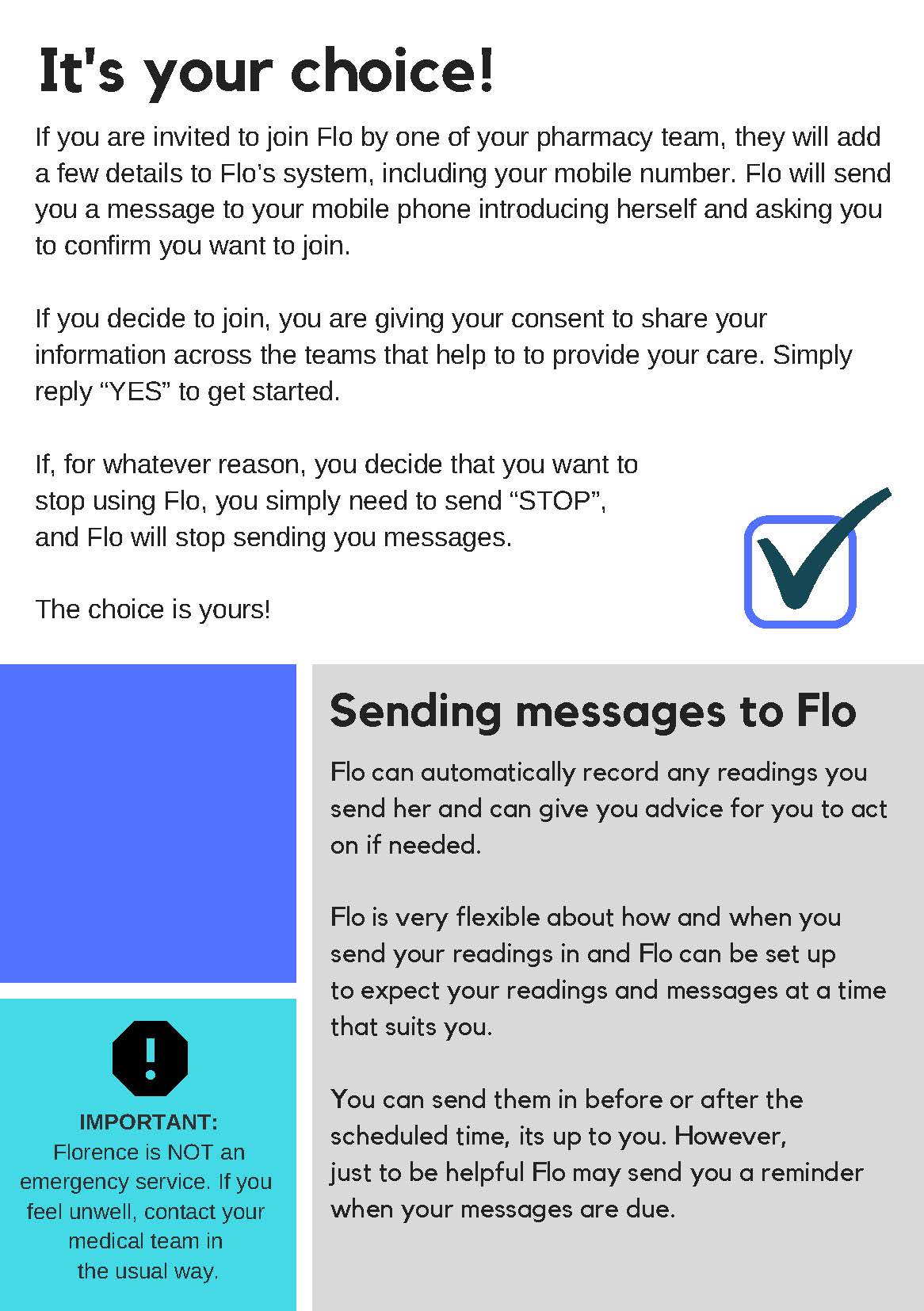

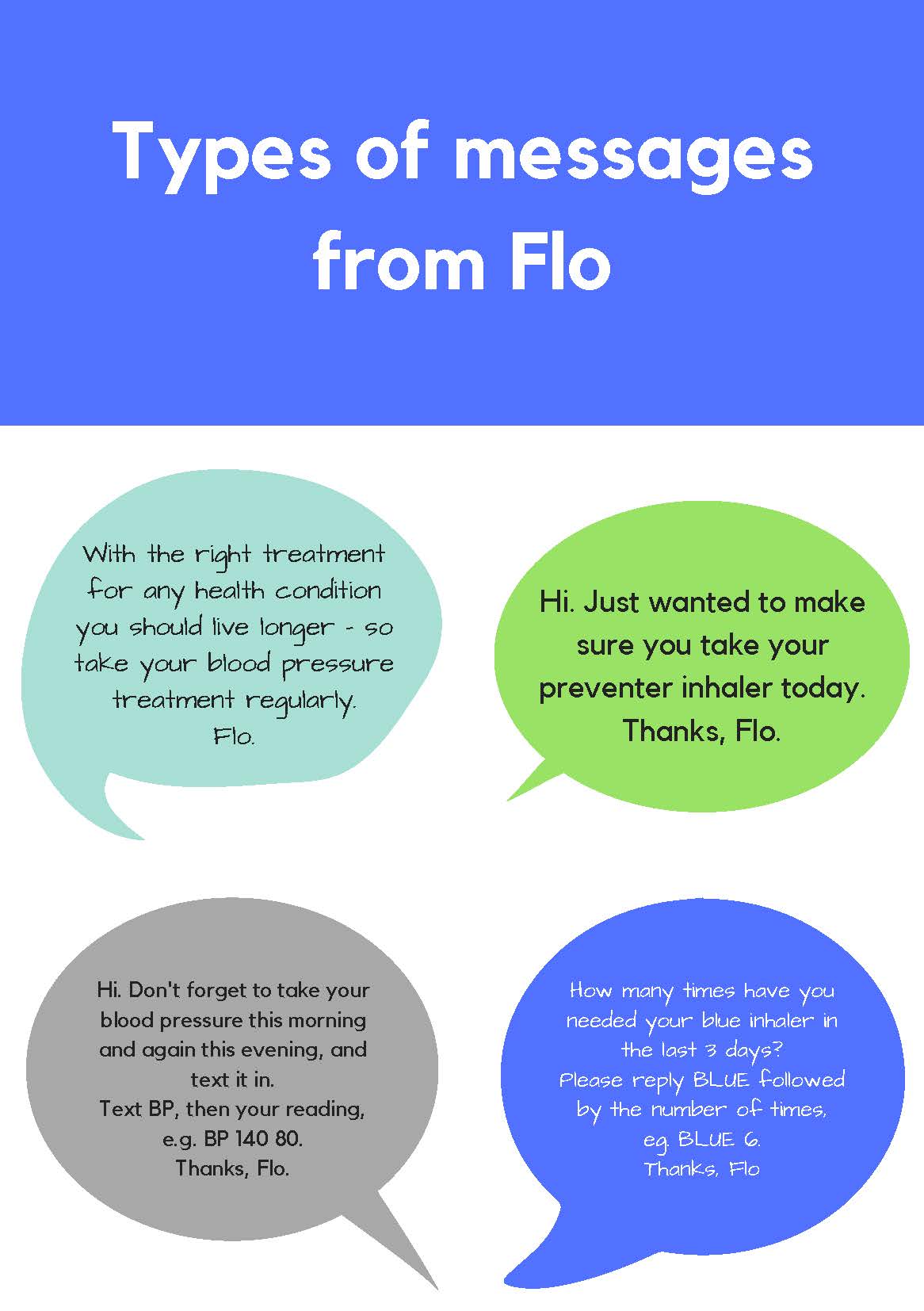

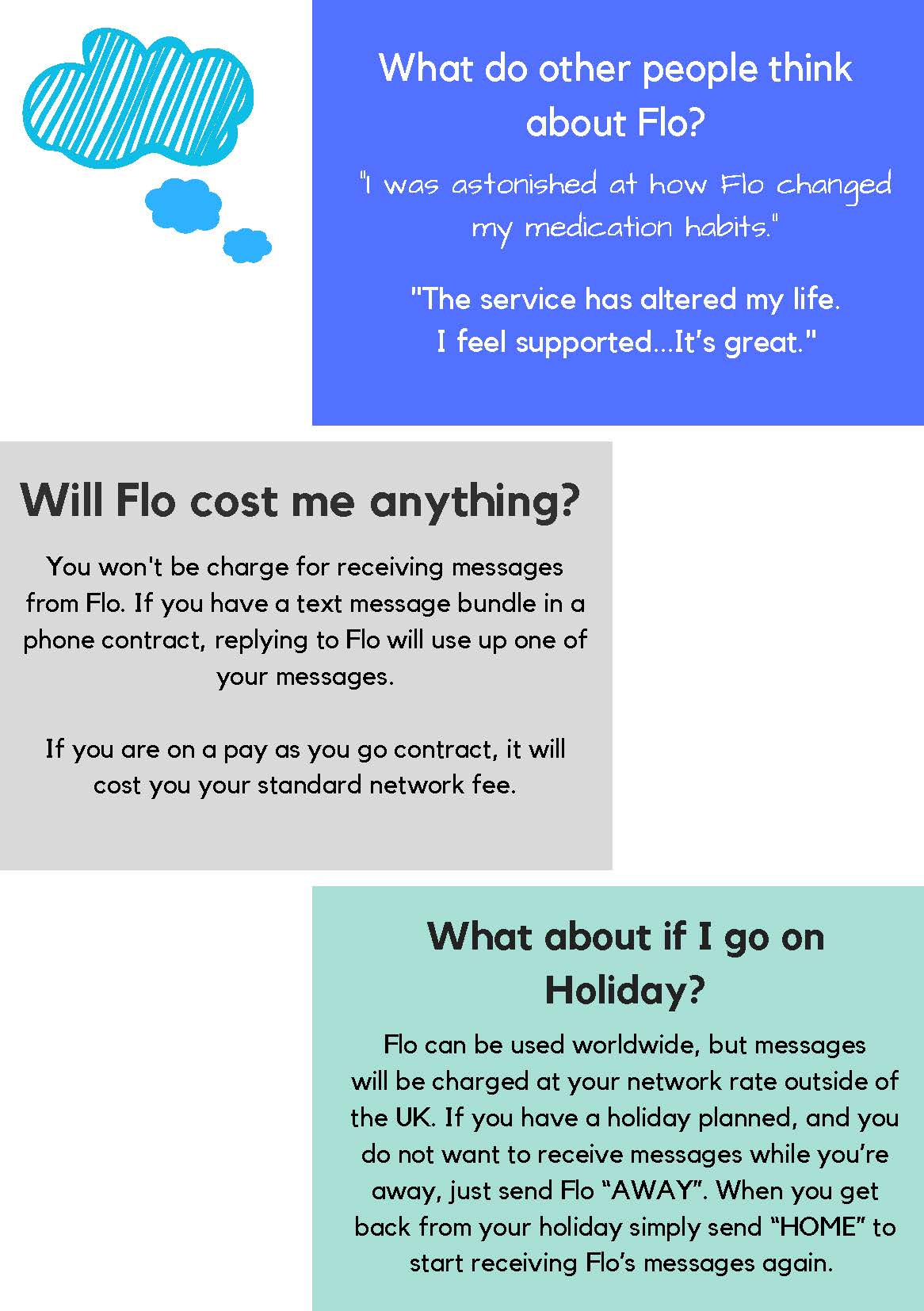

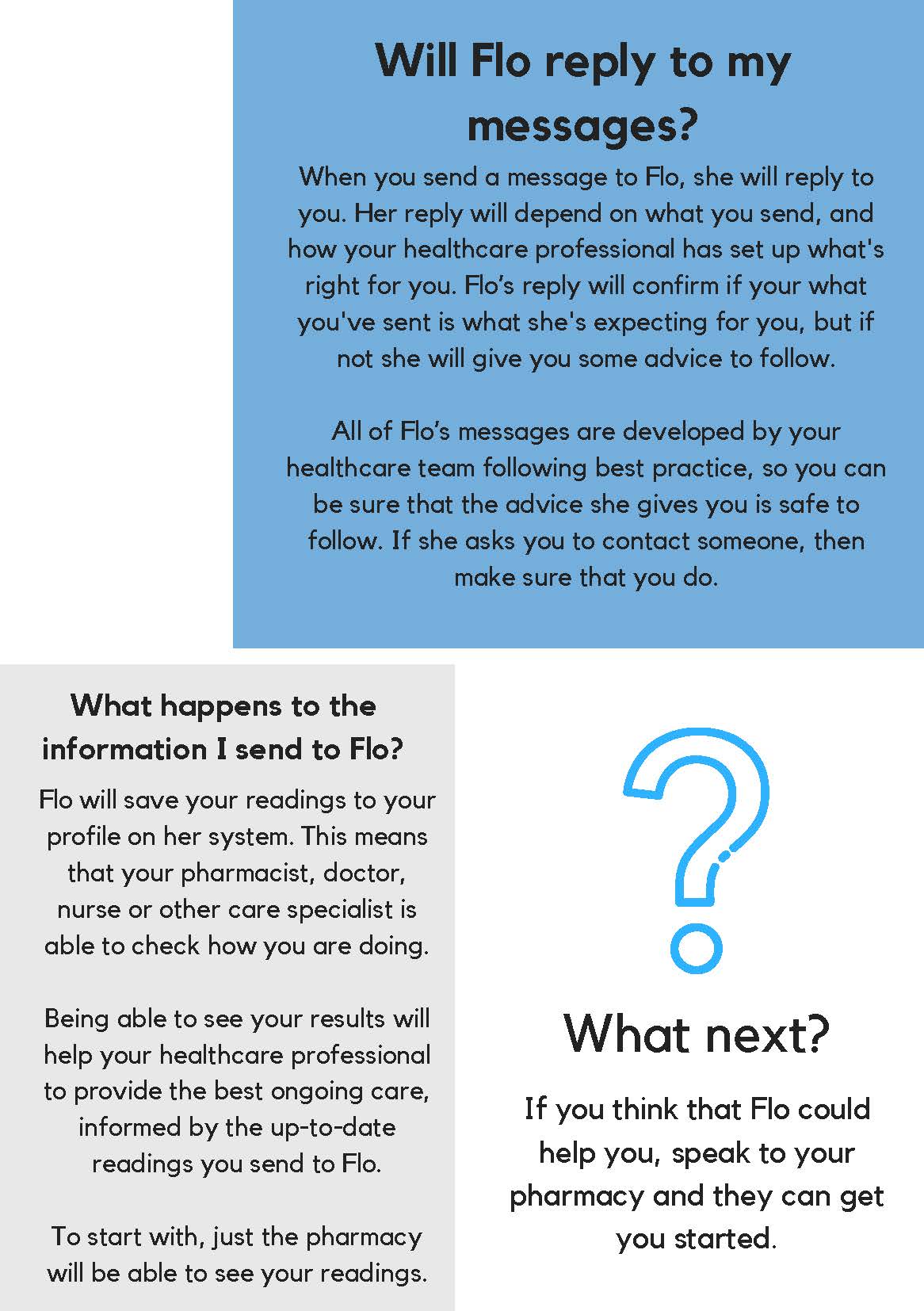


Appendix 2 Collaboration diagram prototype


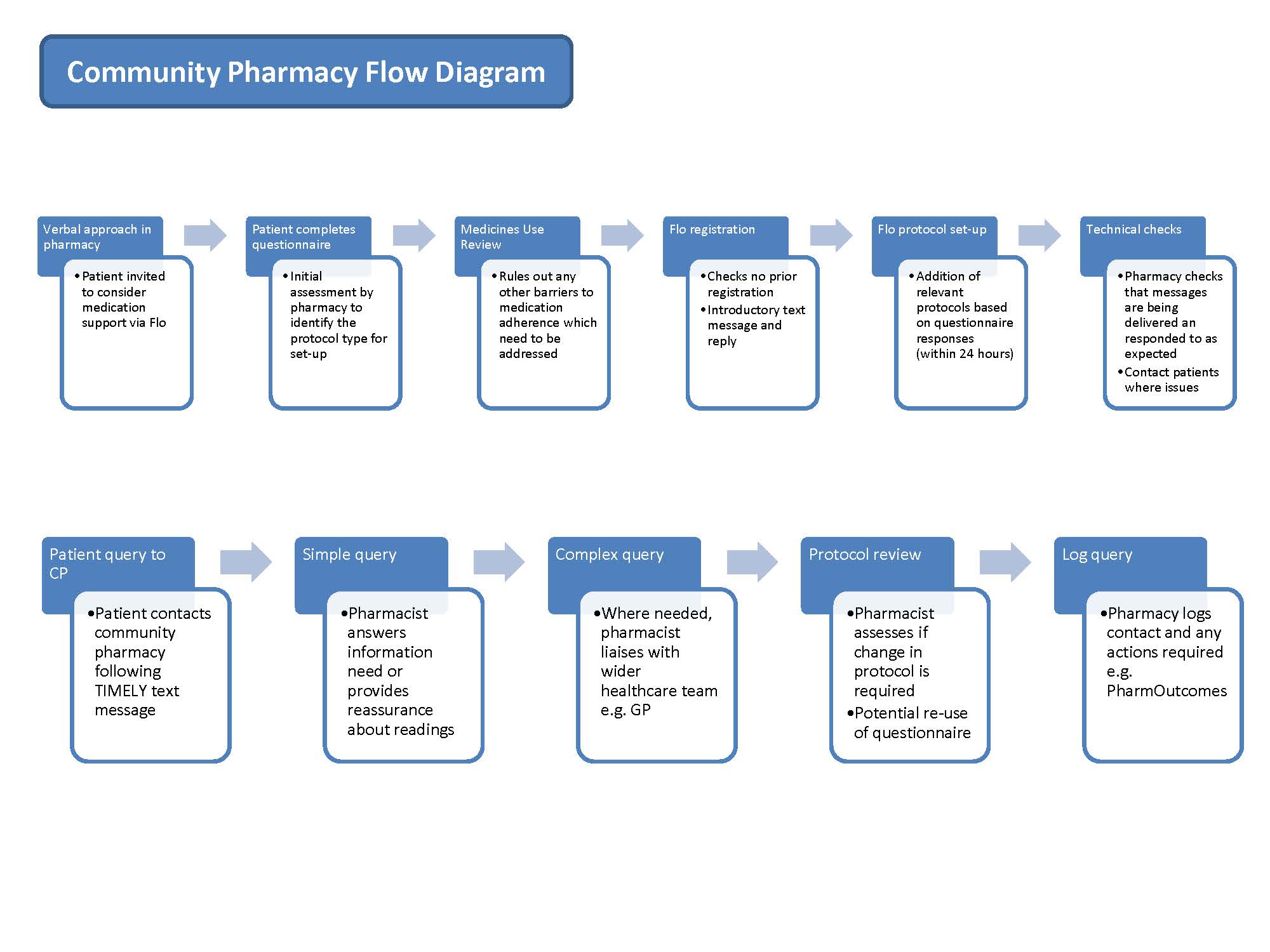

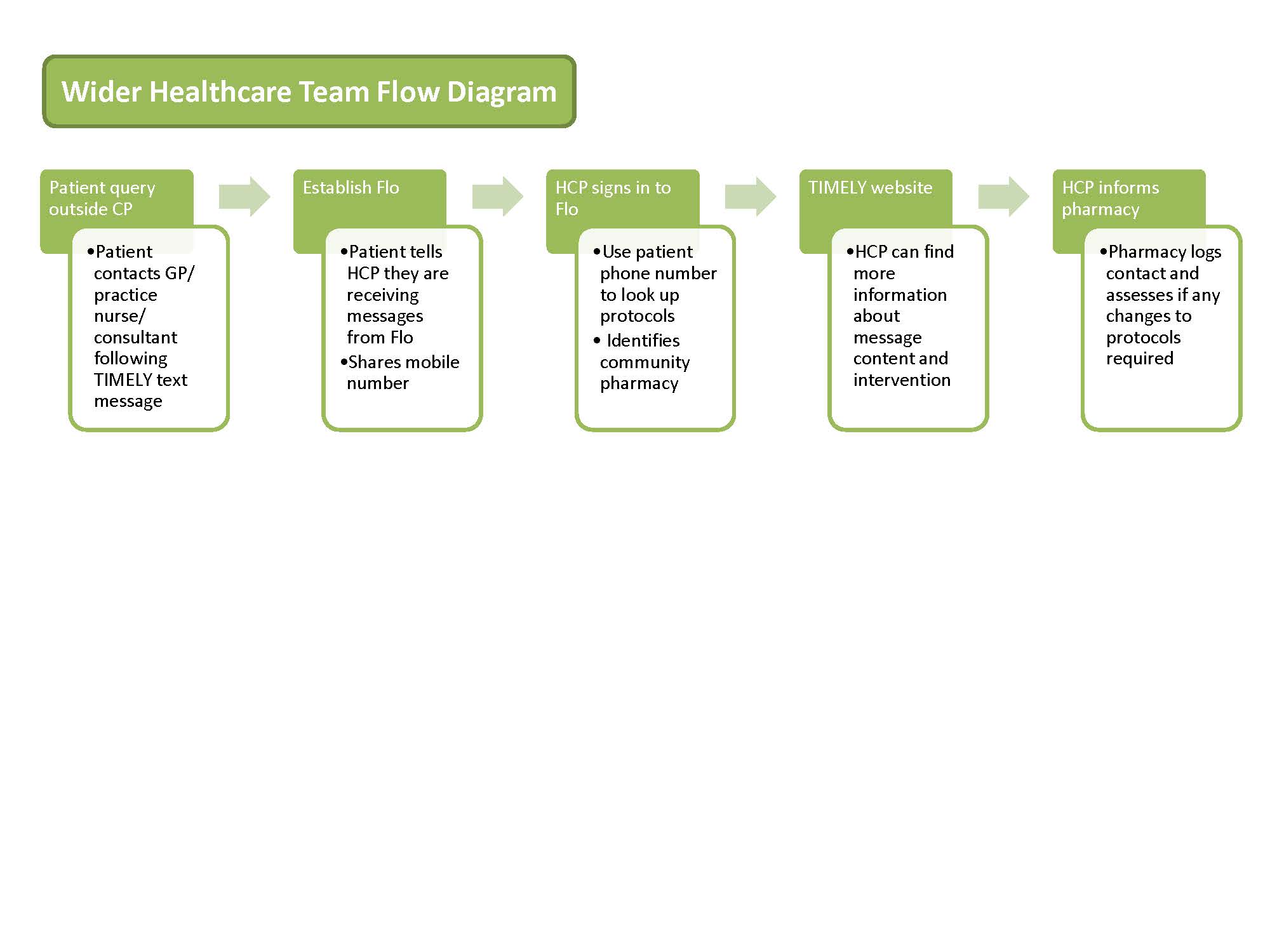

Supplement: Multimedia Appendix 2 [file formative_v6i12e41735_app2.docx]
