# Supplementary material for: Two-way Automated Text Messaging Support From Community Pharmacies for Medication Taking in Multiple Long-term Conditions: Human-Centered Design With Nominal Group Technique Development Study
Source: JMIR Form Res. 2022 Dec 21;6(12):e41735. doi: 10.2196/41735 (PMC9813818; doi:10.2196/41735)

*Multimedia Appendix 3: Ranking questionnaires for the paper: Two-way Automated Text Messaging Support From Community Pharmacies for Medication Taking in Multiple Long-term Conditions: Human-Centered Design With Nominal Group Technique Development Study*
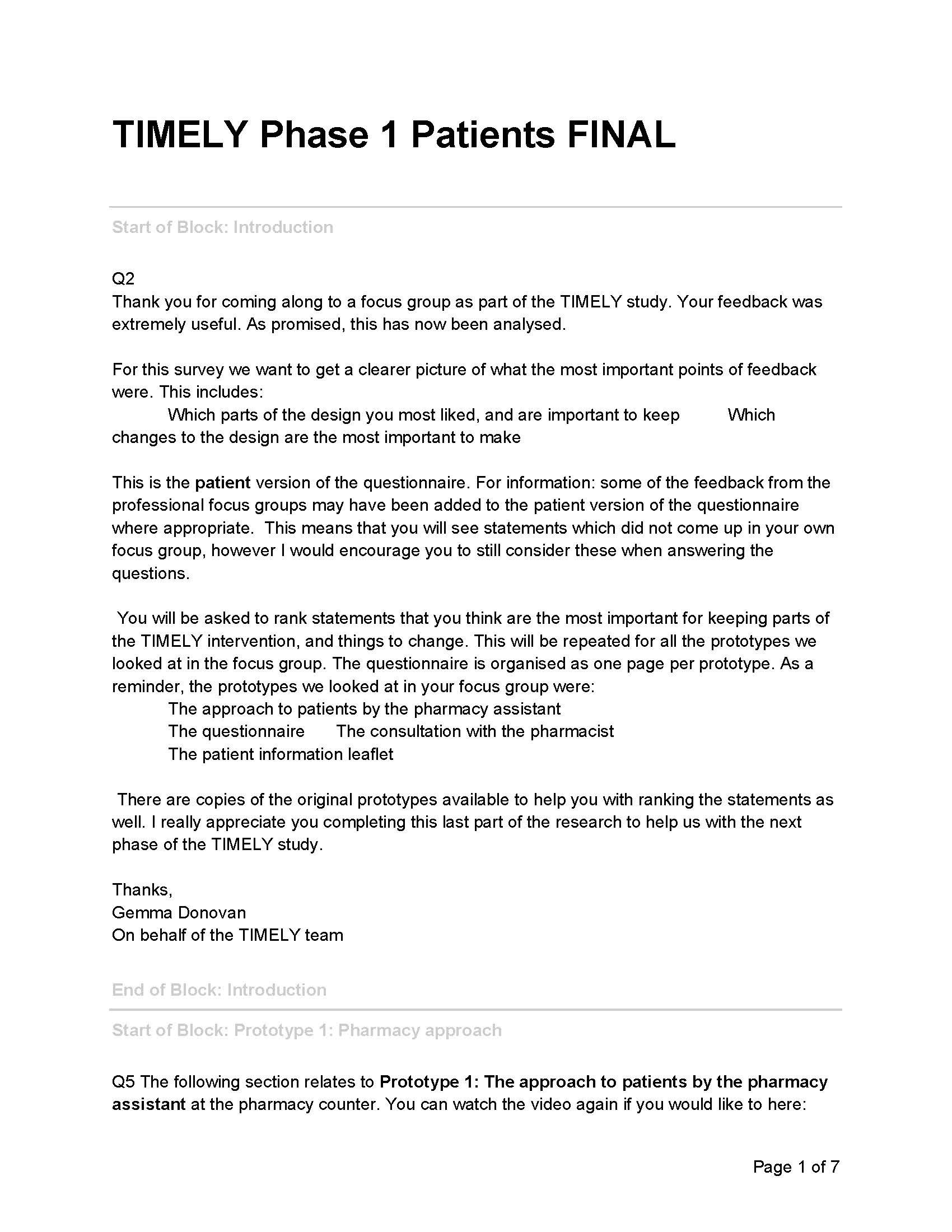

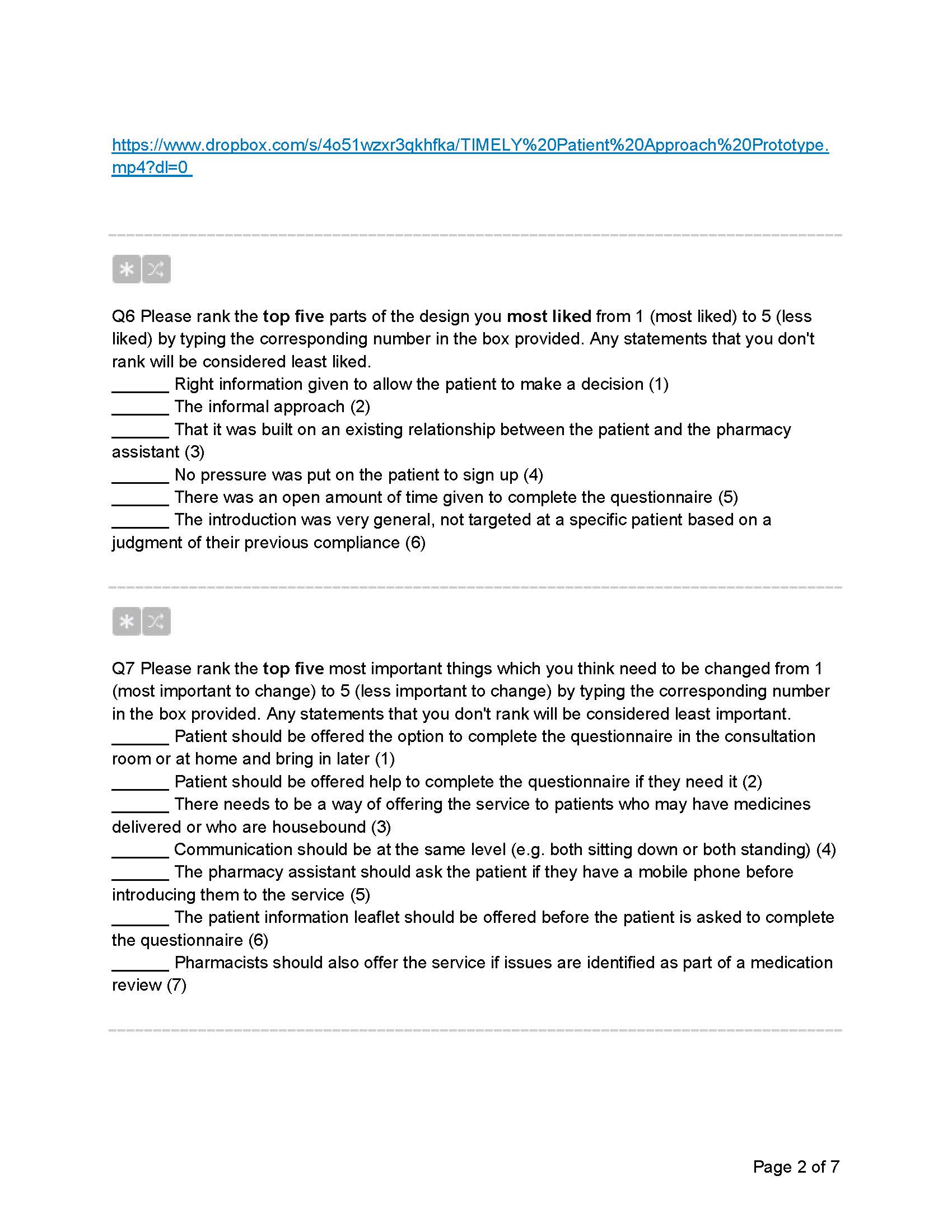

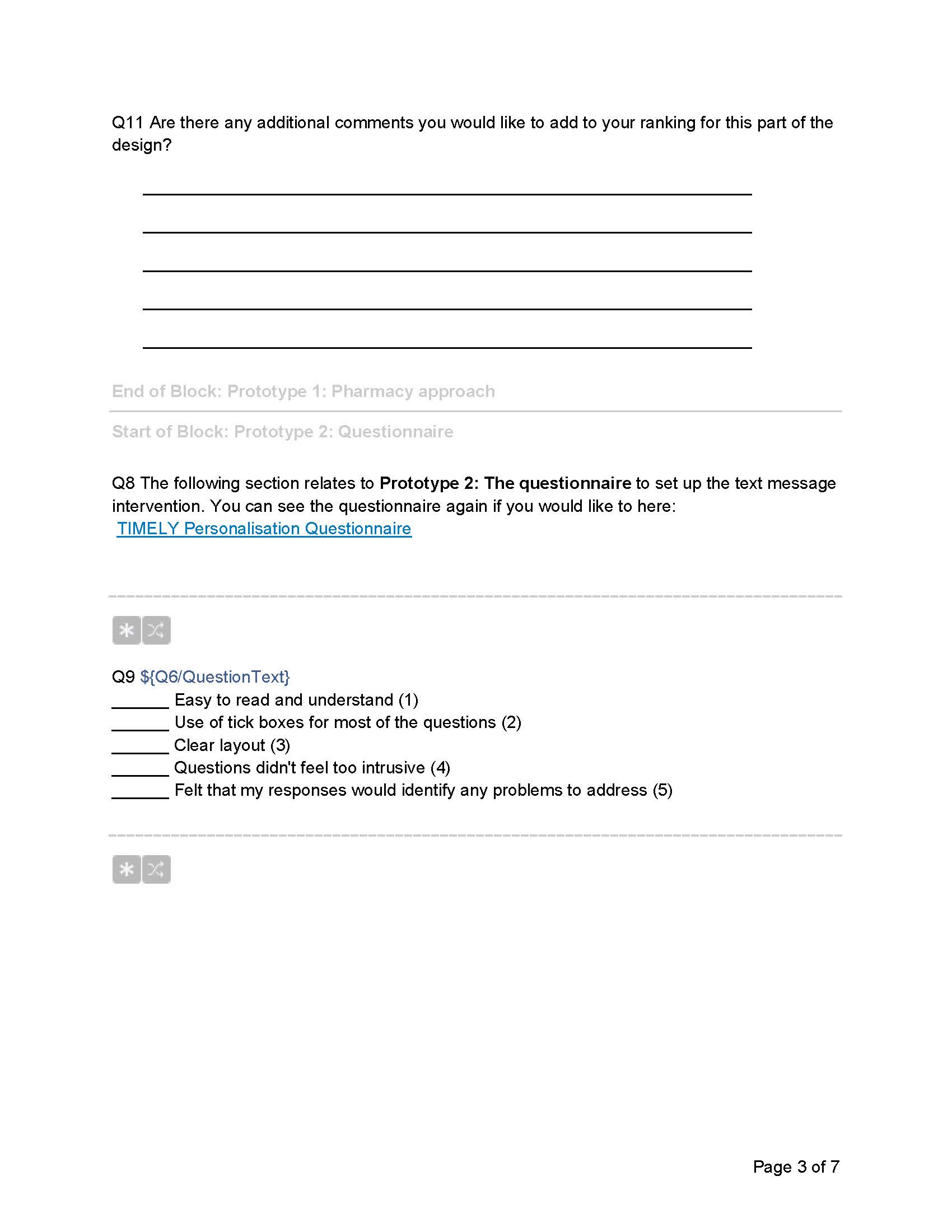

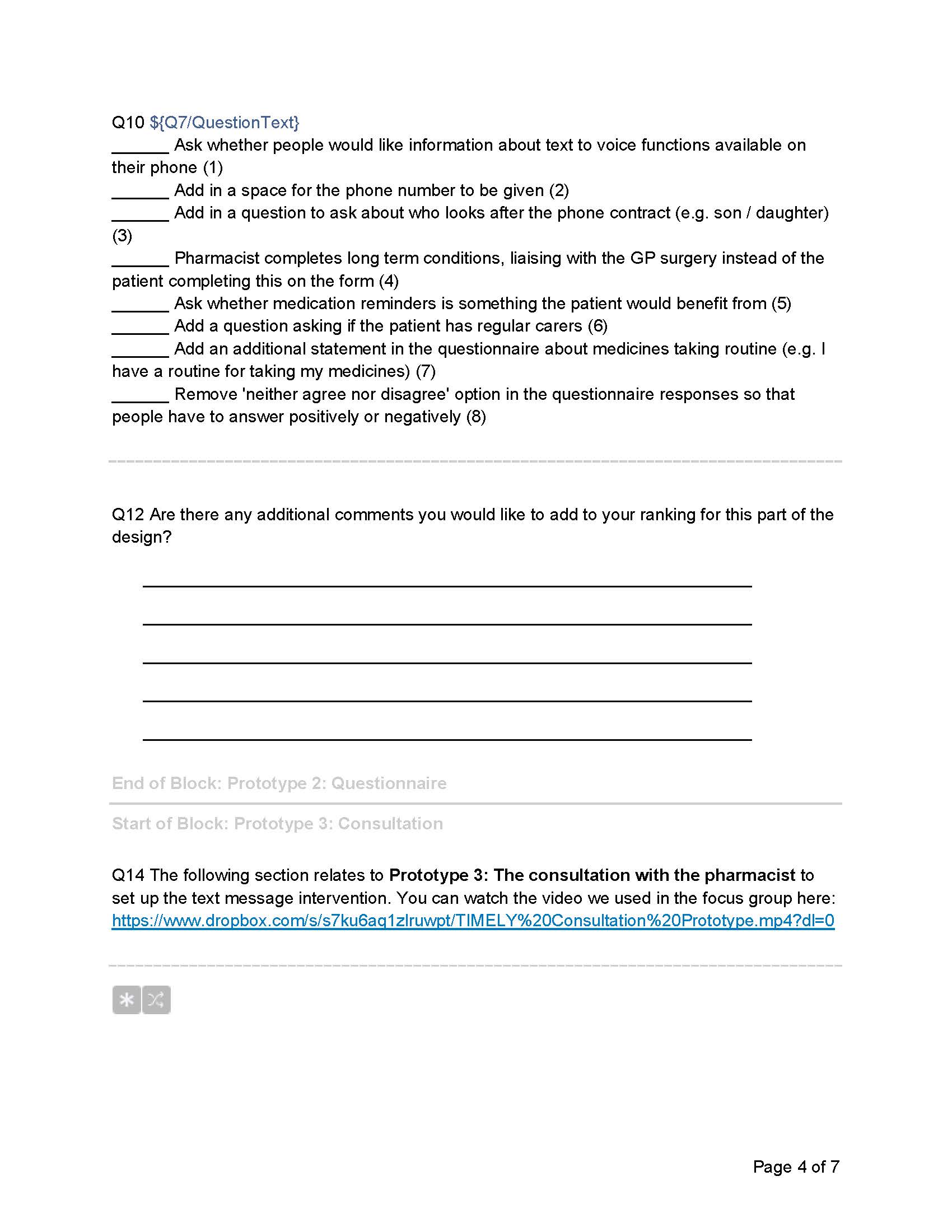

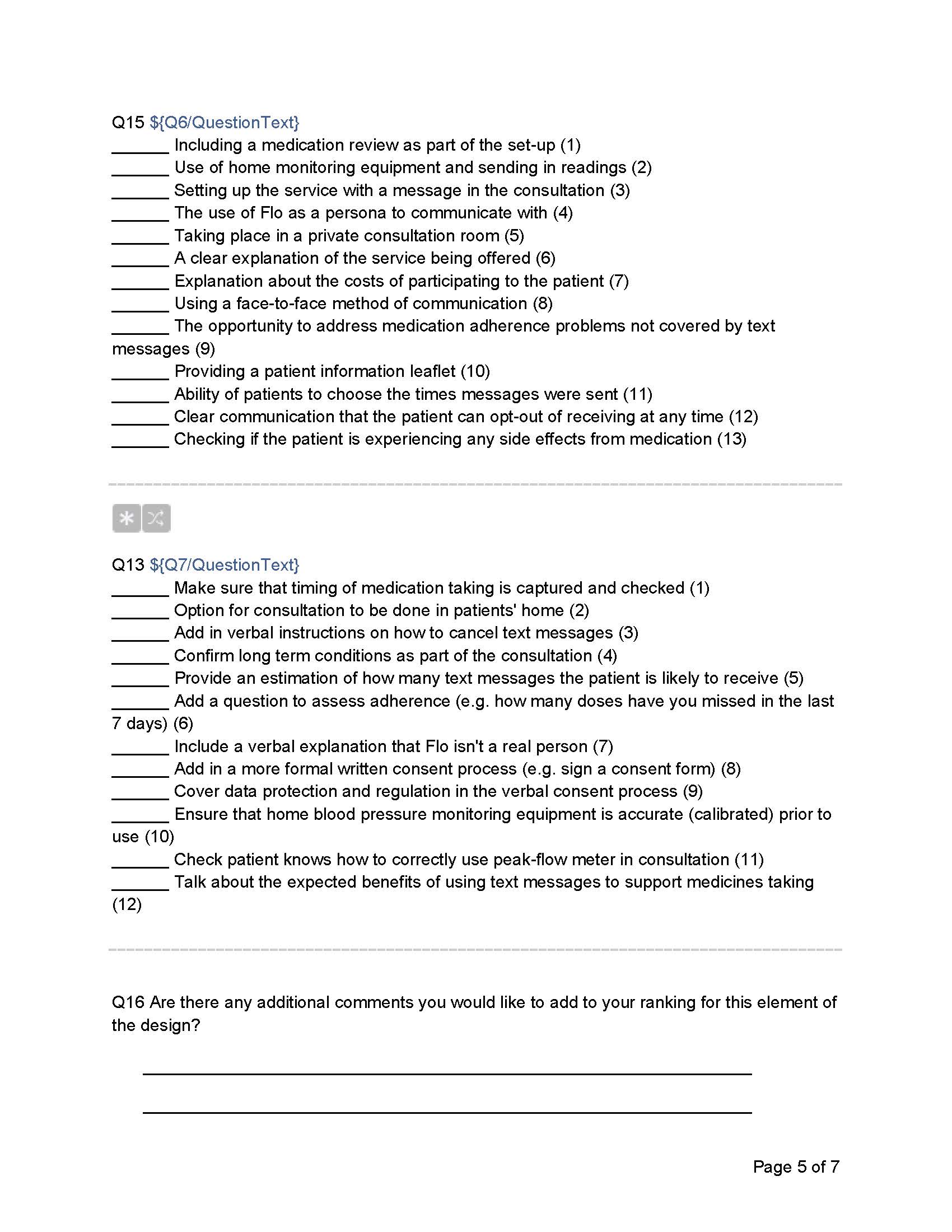

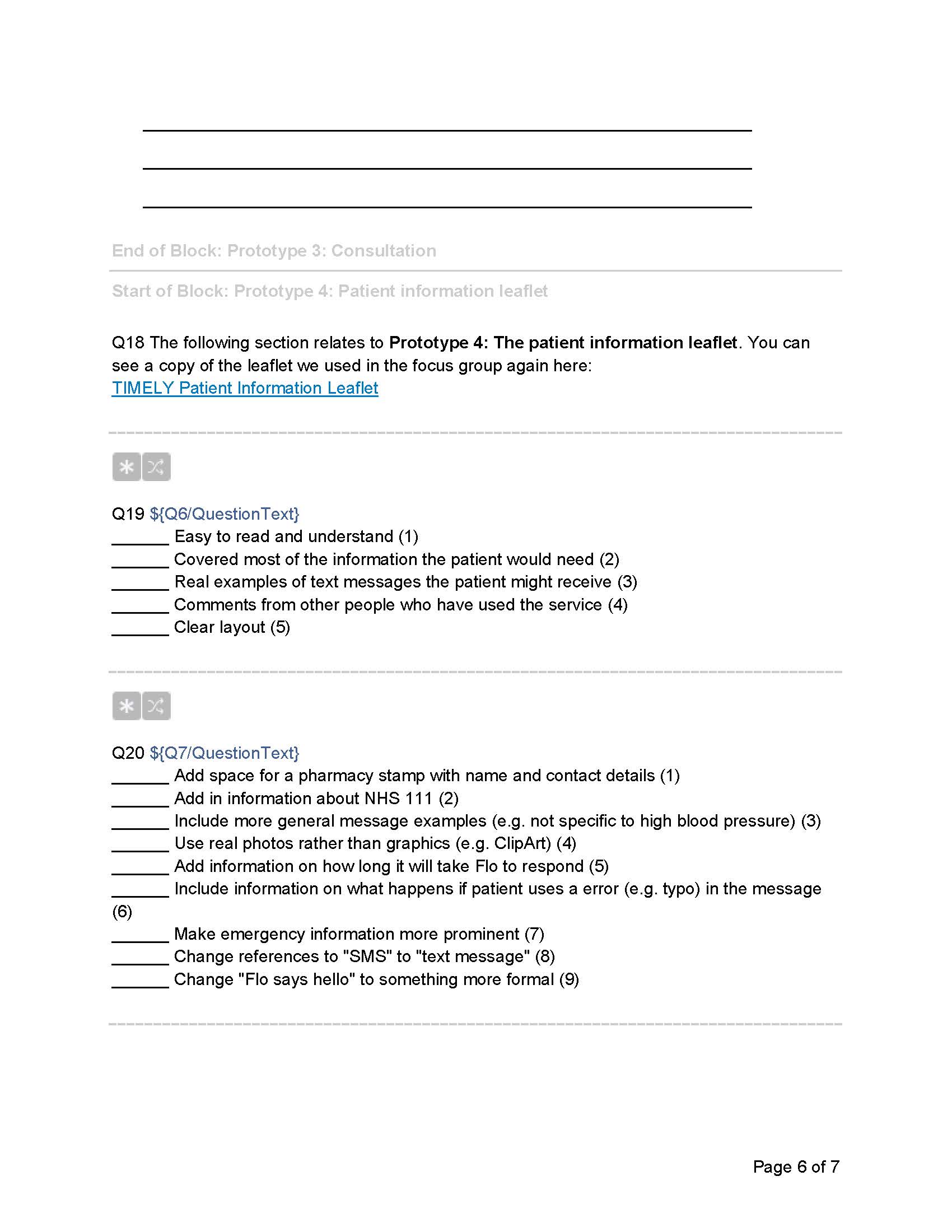

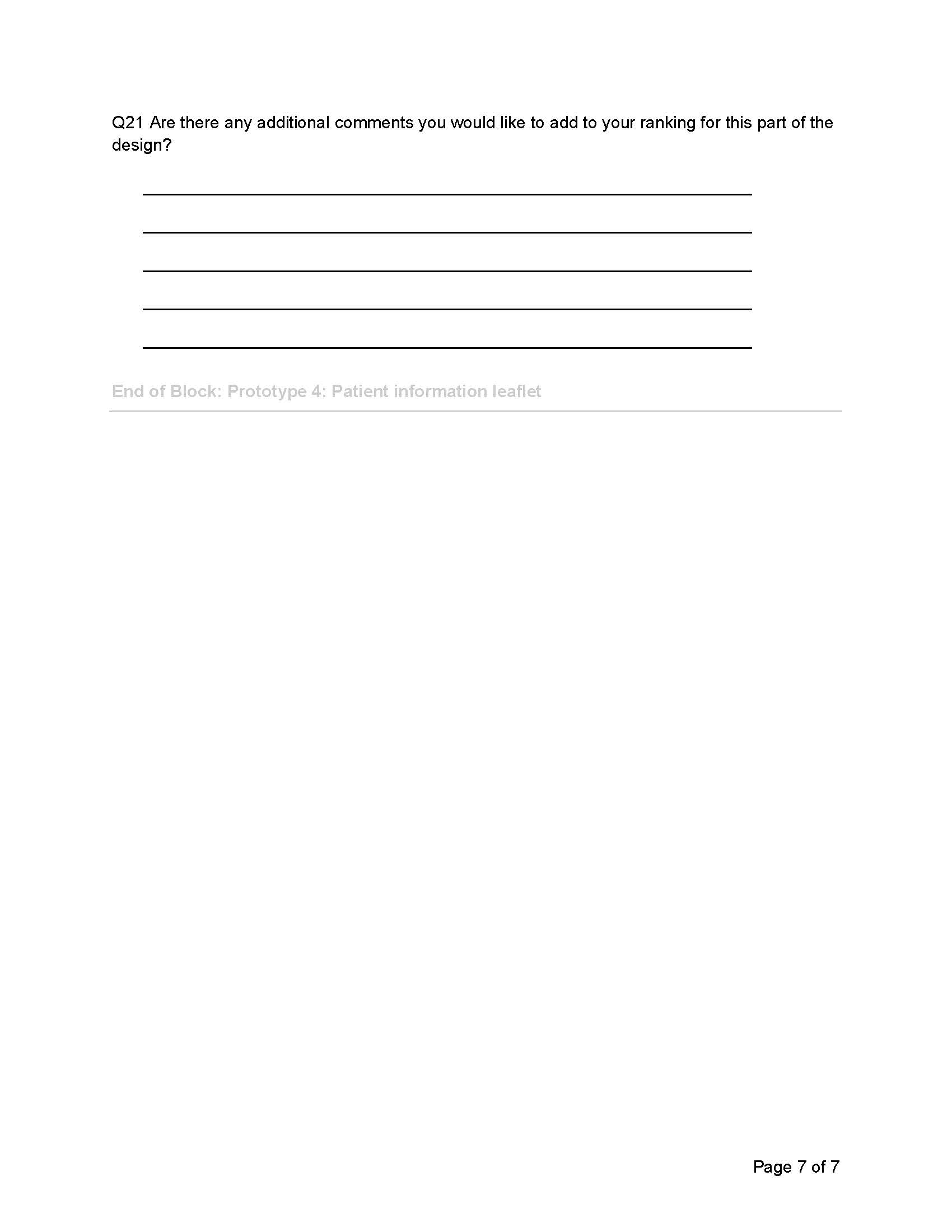

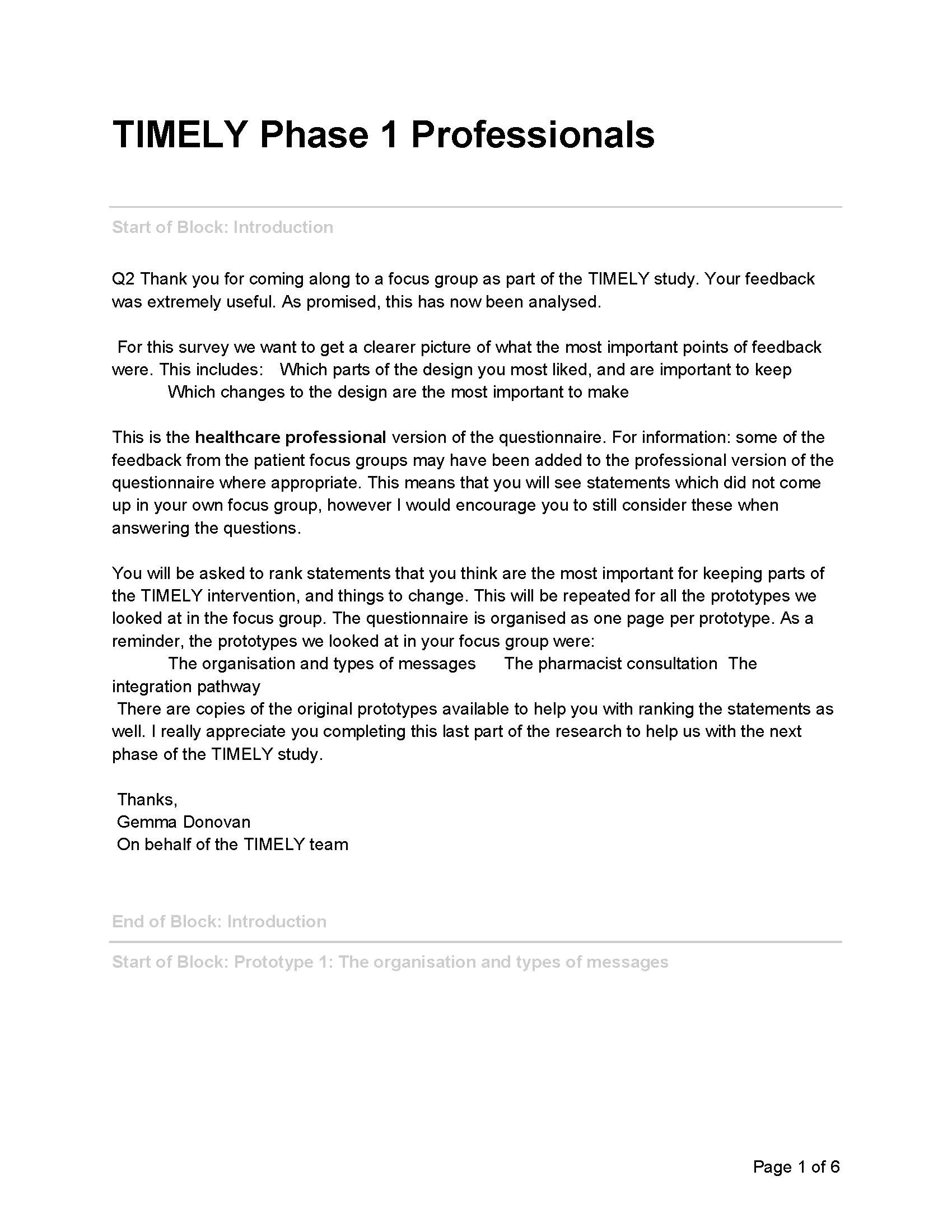

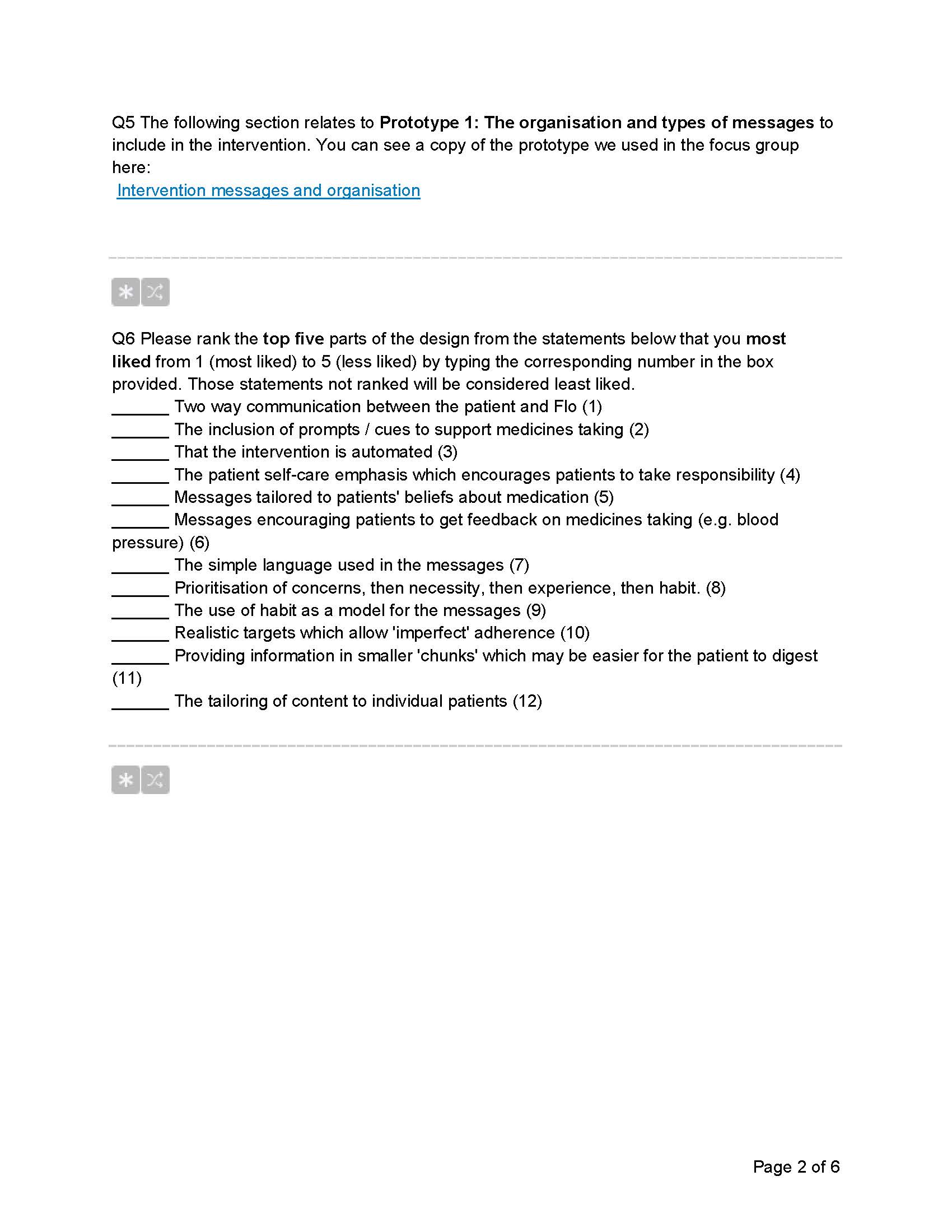

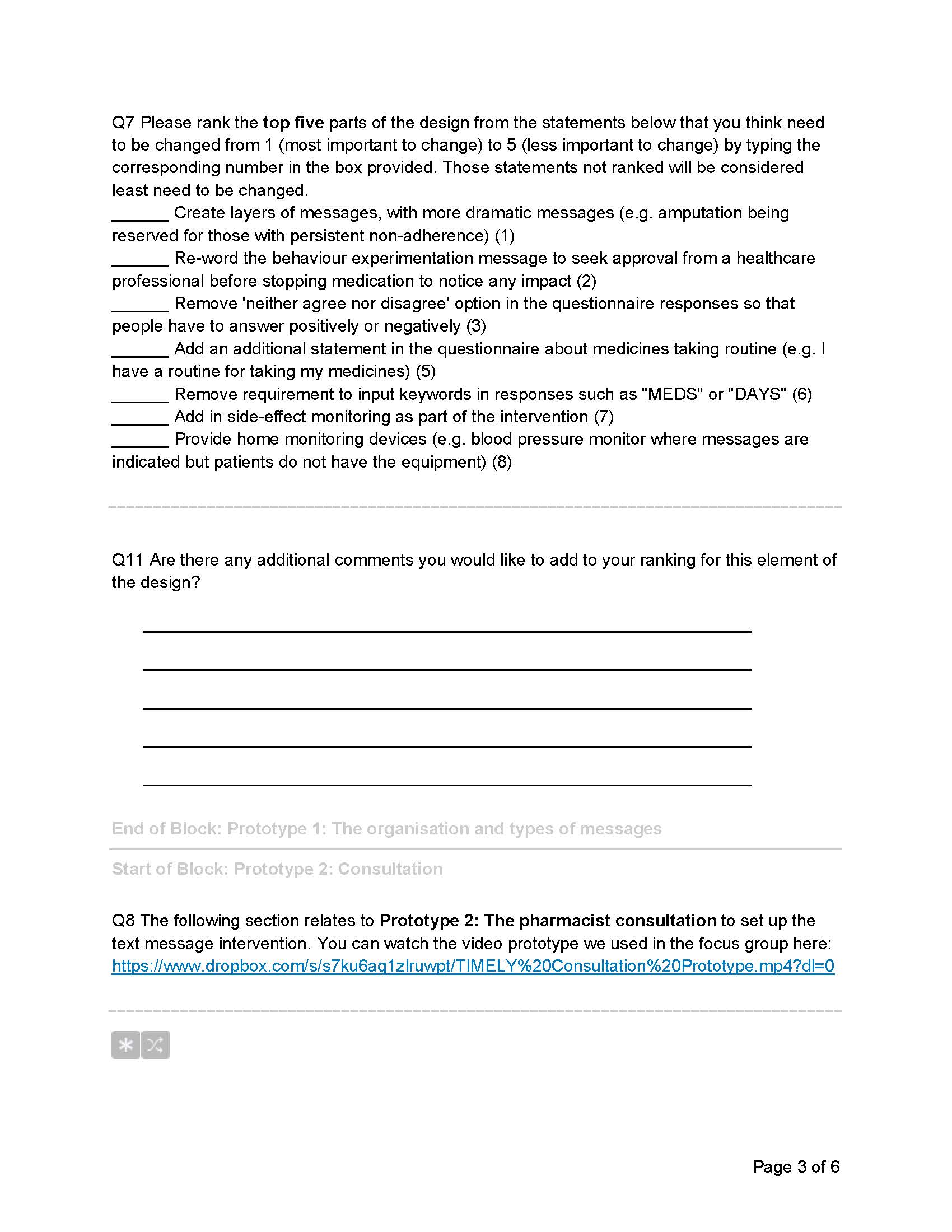

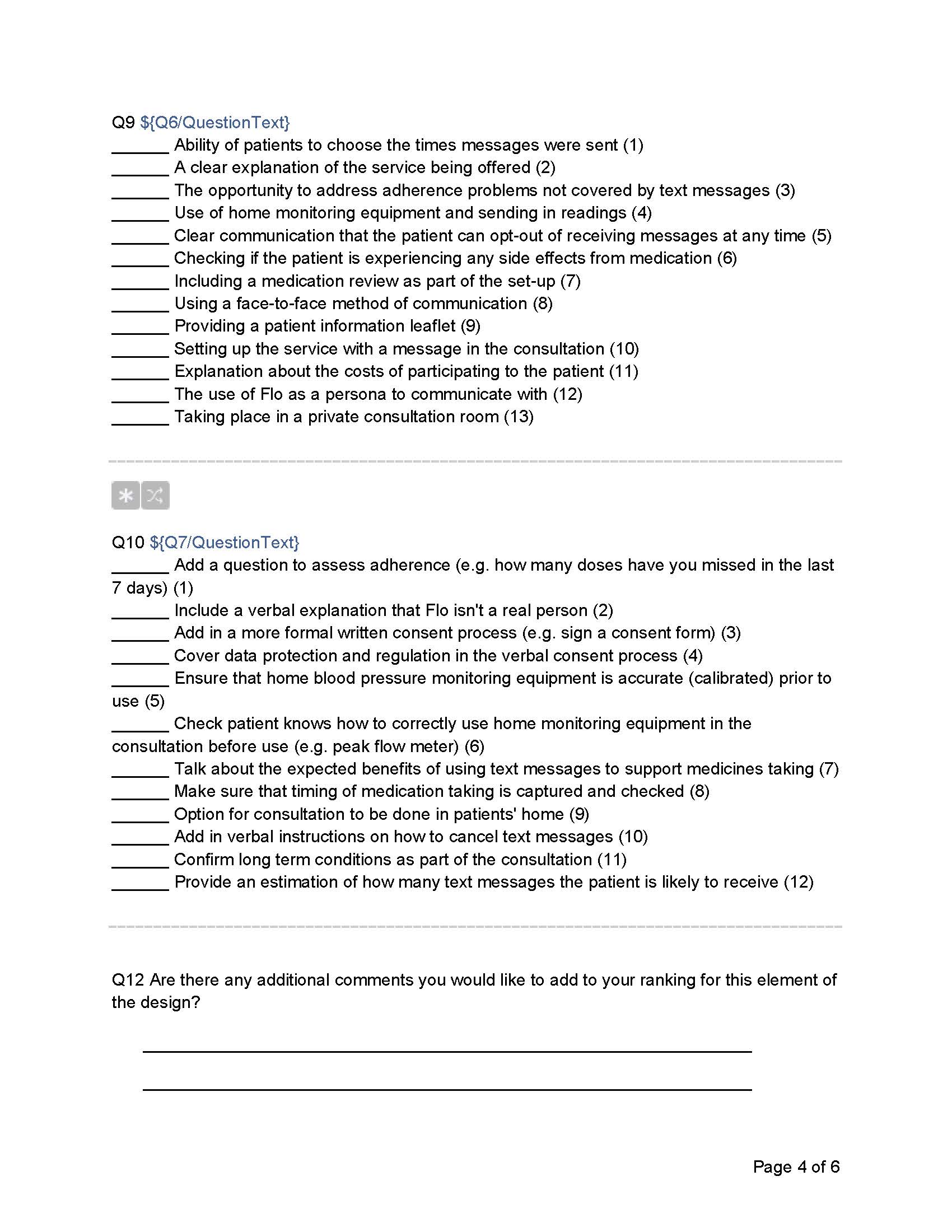

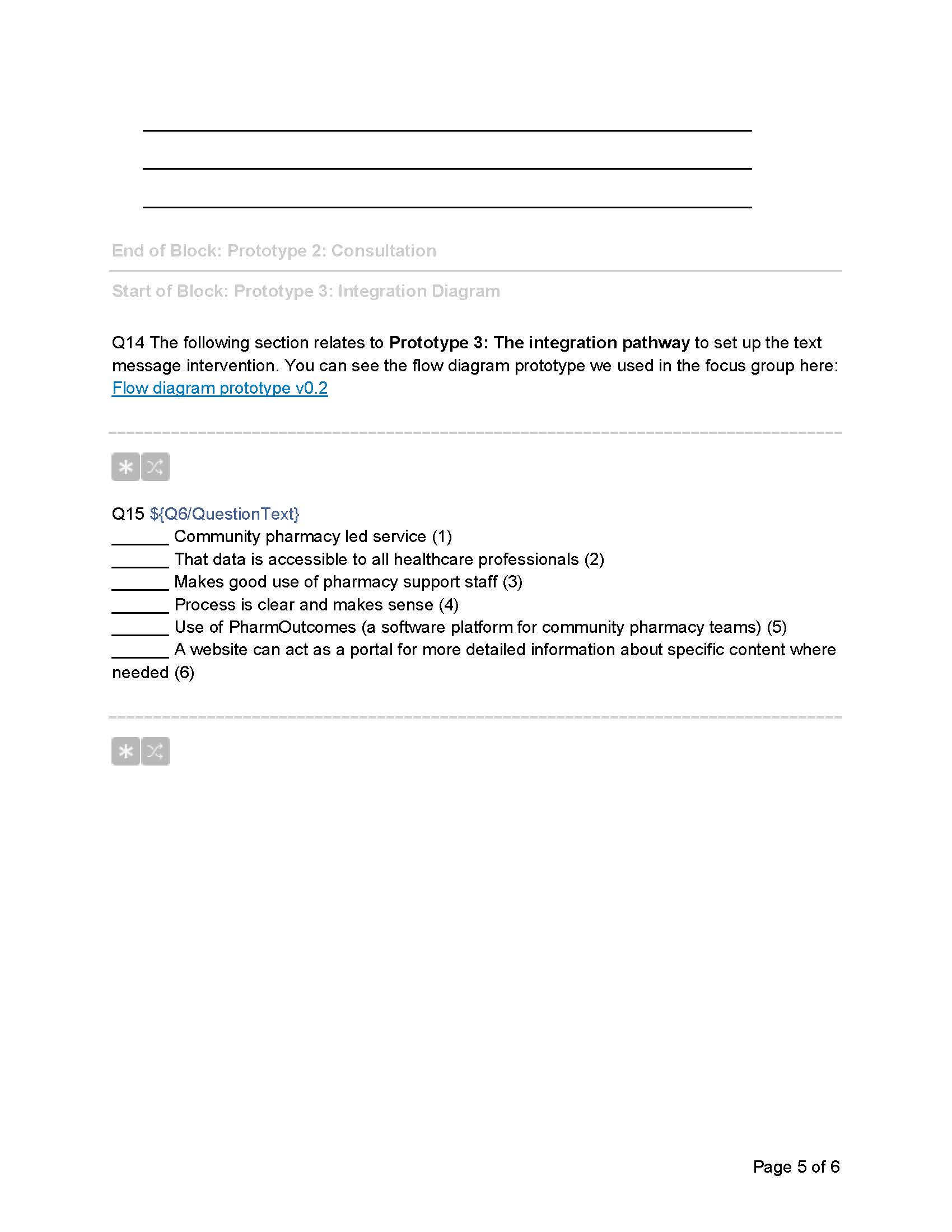

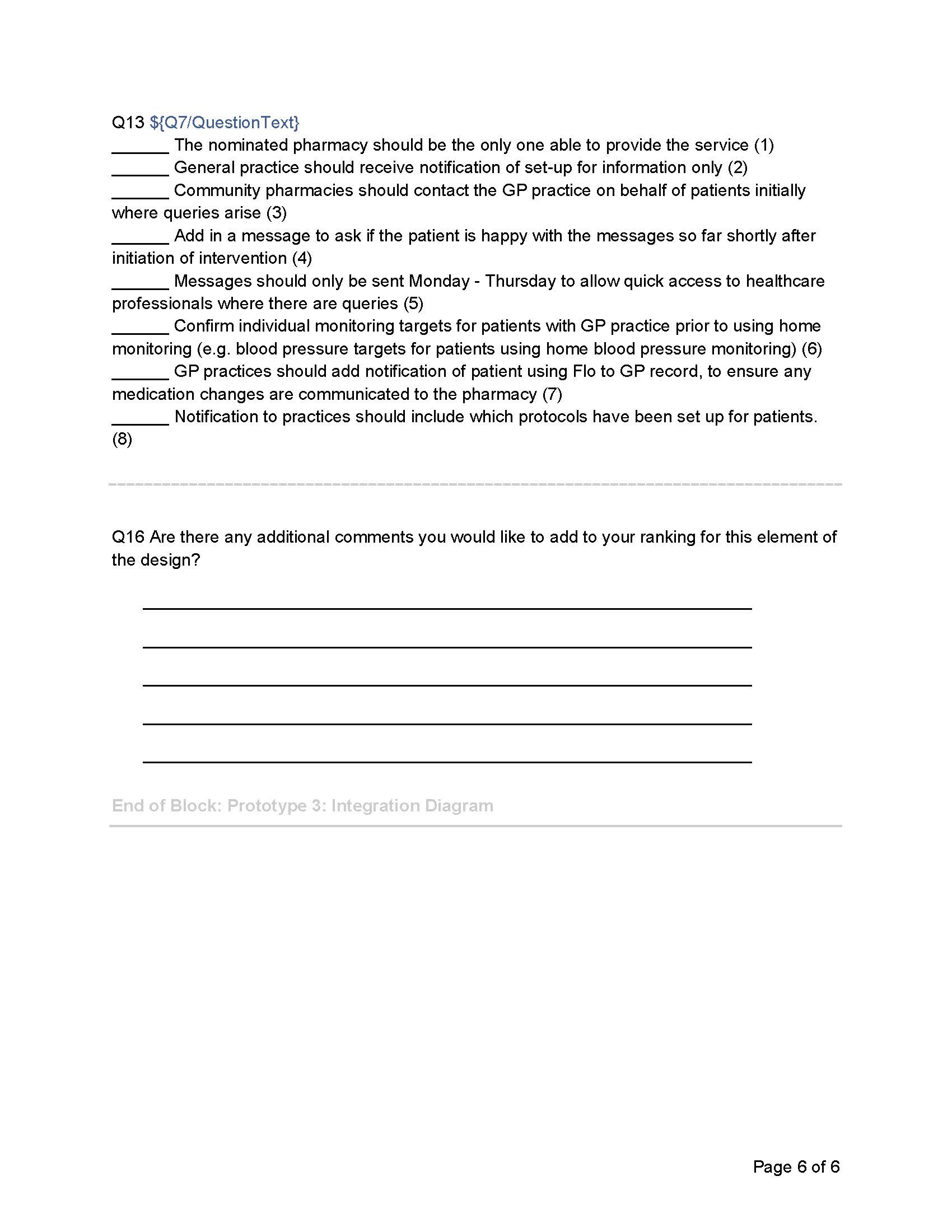

Supplement: Multimedia Appendix 4 [file formative_v6i12e41735_app4.docx]
